# A prospective, randomized, controlled study assessing vagus nerve stimulation using the gammaCore®-Sapphire device for patients with moderate to severe CoViD-19 Respiratory Symptoms (SAVIOR): A structured summary of a study protocol for a randomised controlled trial”

**DOI:** 10.1186/s13063-020-04486-w

**Published:** 2020-06-26

**Authors:** Carlos Tornero, Ricardo Vallejo, David Cedeño, Jorge Orduña, Ernesto Pastor, Moncef Belaouchi, Benigno Escamilla, Marisa Laredo, María del Mar Garzando

**Affiliations:** 1 Hospital Clínico Universitario de Valencia, Anaesthesia, Critical Care and Pain Management Unit, Valencia, Spain; 2 Department of Basic Science, Millennium Pain Center, Bloomington, IL USA; 3 Department of Psychology, Illinois Wesleyan University, Bloomington, IL USA

**Keywords:** COVID-19, Randomised controlled trial, Protocol, Vague neurostimulation, Mechanical ventilation

## Abstract

**Objectives:**

Primary Objective:

The primary objective is to reduce initiation of mechanical ventilator dependency in patients with moderate to severe CoViD- 19. This will be measured as the difference between the control group and active group for subjects admitted to the hospital for CoViD-19.

Secondary Objectives:

• To evaluate cytokine trends / Prevent cytokine storms

• To evaluate supplemental oxygen requirements

• To decrease mortality of CoViD-19 patients

• Delay onset of ventilation

**Trial design:**

The study is a single centre, 2-arm, prospective, randomized (ratio 1:1), controlled trial with parallel groups design to compare the reduction of respiratory distress in a CoViD-19 population, using the intervention of the gammaCore®-Sapphire device plus standard of care (active) vs. standard of care alone (SoC) - the control group. The gammaCore® treatments will be used acutely and prophylactically. The active and control groups will be matched for disease and severity.

**Participants:**

**i. Inclusion Criteria**

The subjects have to meet all of the following criteria to be eligible to enter the trial:

1. Patient older than 18 years
2. Been tested positive or suspected/presumed positive for CoViD-19

Has a cough, shortness of breath or respiratory O_2_ Saturation less than or equal to 92% without need for mechanical ventilation or acute respiratory failure

3. Agree to use the gammaCore®-Sapphire device as intended and to follow all of the requirements of the study including recording required study data
4. Patient is able to provide signed and witnessed Informed Consent

**ii. Exclusion Criteria**

Subjects meeting any of the following criteria cannot be included in this research study:

1. Pregnant women
2. On home/therapy oxygen (i.e. for patients with Chronic Obstructive Pulmonary Disease) at baseline prior to development of CoViD-19
3. Patient already enrolled in a clinical trial using immunotherapeutic regimen for CoViD-19
4. History of aneurysm, intracranial hemorrhage, brain tumors, or significant head trauma
5. Known or suspected severe atherosclerotic cardiovascular disease, severe carotid artery disease (eg, bruits or history of transient ischemic attack or cerebrovascular accident), congestive heart failure, known severe coronary artery disease, or recent myocardial infarction
6. Uncontrolled high blood pressure (>140/90)
7. Current implantation of an electrical and/or neurostimulator device, including but not limited to a cardiac pacemaker or defibrillator, vagal neurostimulator, deep brain stimulator, spinal stimulator, bone growth stimulator, or cochlear implant
8. Current implantation of metal cervical spine hardware or a metallic implant near the gammaCore stimulation site
9. Belongs to a vulnerable population or has any condition such that his or her ability to provide informed consent, comply with the follow-up requirements, or provide self-assessments is compromised (e.g. homeless, developmentally disabled and prisoner)

Participants will be recruited from Hospital Clínico Universitario de Valencia in Spain.

**Intervention and comparator:**

Intervention:

- Prophylactic: Administer 2 doses (at 2 minutes each) of gammaCore®^-^Sapphire, one dose on each side of the neck scheduled three times a day (morning, mid-day and 1 hour before bed at night).
- Acute respiratory failure or shortness of breath: Administer 2 doses (at 2 minutes each) of gammaCore®-Sapphire, one on each side of the neck. If shortness of breath (SOB) persists 20 minutes after the start of the first treatment, a second dose will be administered. Max doses per day is 9 or 18 stimulations.
- Plus standard of care

Control:

- Standard of care: oxygen therapy, antibiotics and ventilatory support if necessary depending on the clinic

**Main outcomes:**

Primary Endpoint:

- Initiation of mechanical ventilation, from randomization until ICU admission or hospital discharge, whatever occurs first

Secondary Endpoints:

- Safety; ascertainment of Adverse Effects/Serious Adverse Events, from randomisation to ICU admission or hospital discharge, whatever occurs first
- Cytokine Storm measured by: Tumor necrosis factor α, Interleukin 6, Interleukin 1β. Days 1,3,5,10,15 and/or at hospital discharge
- Mortality and/or need for Critical Care admission, from randomisation until ICU admission or hospital discharge, whatever occurs first,
- O2 saturation levels , from randomization until ICU admission or hospital discharge, whatever occurs first
- Need for supplemental oxygen, from randomisation until ICU admission or hospital discharge, whatever occurs first

**Randomisation:**

The patients are classified according to their oxygen levels as mild, moderate and severe and randomized according to their classification to the intervention and control in a ratio of 1:1. The randomization will be stratified for gender and age.

**Blinding (masking):**

This is an open label study, it is not possible to blind the participants and healthcare providers to the intervention.

**Numbers to be randomised (sample size):**

The total number of patients to be included in the study is 90, with 45 in each study group

**Trial Status:**

The protocol version is 8.0 from 07^th^ April 2020. The recruitment began 20th April 2020 and is expected to be complete 31st July 2020.

**Trial registration:**

The study is registered in clinicaltrials.gov on 29th April 2020 with the identification number: NCT04368156

**Full protocol:**

The full protocol is attached as an additional file, accessible from the Trials website (Additional file 1). In the interest in expediting dissemination of this material, the familiar formatting has been eliminated; this Letter serves as a summary of the key elements of the full protocol.

## Supplementary information

**Additional file 1.**

## Data Availability

Not applicable.

