# Supplementary material for: A prospective, randomized, controlled study assessing vagus nerve stimulation using the gammaCore®-Sapphire device for patients with moderate to severe CoViD-19 Respiratory Symptoms (SAVIOR): A structured summary of a study protocol for a randomised controlled trial”
Source: Trials. 2020 Jun 26;21:576. doi: 10.1186/s13063-020-04486-w (PMC7316576; doi:10.1186/s13063-020-04486-w)
Supplement: Supplementary file 1 — Additional file 1. [file 13063_2020_4486_MOESM1_ESM.pdf]

CONFIDENCIAL

PLAN DE INVESTIGACION CLINICA (PIC)

**Título:** Estudio Prospectivo, Aleatorizado, Controlado para Evaluar el Efecto de la Estimulación Eléctrica No-Invasiva del Nervio Vago en Síntomas Respiratorios debido a CoViD-19 (SAVIOR)	
Codigo del Plan de Investigacion Clinica:	SAVIOR
Dispositivo Medico a utilizar en la Investigacion Clinica:	gammaCore®-Sapphire
Indicacion:	Sindrome de insuficiencia respiratoria aguda
Clase del Dispositivo:	IIa
Version del Protocol:	8.0
Fecha:	7 de abril de 2020
Promotor e Investigador Principal/Coordinador del Proyecto	Dr. Carlos Tornero, M.D.<br>Jefe Servicio Anestesiología-Reanimación<br>Segunda Planta Unidad Reanimación<br>Hospital Clínico Universitario de Valencia<br>Avenida Blasco Ibáñez 17<br>46010 Valencia, España.
Propietario del dispositivo:	electroCore LLC<br>150 Allen Road, Suite 201<br>Basking Ridge, NJ 07920<br>USA

Esta investigación clínica se hará en conformidad con la ISO 14155:2011(E), las regulaciones para dispositivos médicos (Medical Device Directive, MDD), la Declaración de Helsinki y todas las regulaciones que apliquen.

CONFIDENCIAL
Este Plan de Investigación Clínica contiene información privilegiada o confidencial, la cual es propiedad del Promotor. Tal información no puede compartirse con terceros sin previa autorización escrita del Promotor.

Esta investigación clínica se hará en conformidad con la ISO 14155:2011(E), las regulaciones para dispositivos médicos (Medical Device Directive, MDD), la Declaración de Helsinki y todas las regulaciones que apliquen.

CONFIDENCIAL
Este Plan de Investigación Clínica contiene información privilegiada o confidencial, la cual es propiedad del Promotor. Tal información no puede compartirse con terceros sin previa autorización escrita del Promotor.

1 SYNOPSIS

**PROPIETARIO DEL DISPOSITIVO:**<br>Los dispositivos médicos son suministrados por electroCore LLC
**CODIGO DE INVESTIGACION CLINICA:**<br>SAVIOR
**TITULO DE LA INVESTIGACION CLINICA:** Estudio Prospectivo, Aleatorizado con Grupo de Control para Evaluar el Efecto de la Estimulación Eléctrica No-Invasiva del Nervio Vago en Síntomas Respiratorios debido a CoViD-19 (SAVIOR)
**NOMBRE DEL DISPOSITIVO MEDICO EN LA INVESTIGACION CLINICA:**<br>gammaCore®-Sapphire
**OBJETIVOS:**<br>Objetivo Primario:<br>• Prevenir la necesidad de un ventilador (respirador) mecánico en pacientes con CoViD-19 mediante la utilización de la estimulación no-invasiva del nervio vago utilizando el dispositivo gammaCore®-Sapphire relativo a un grupo de control.<br>Objetivos Secundarios:<br>• Evaluar el nivel de citoquinas circulantes / Prevenir las tormentas de citoquinas inflamatorias<br>• Evaluar los requerimientos de oxigeno<br>• Disminuir la estancia hospitalaria<br>• Disminuir la mortalidad y/o necesidad de cuidados intensivos en pacientes internados en el hospital con CoViD-19<br>• Medir la frecuencia de eventos adversos que puedan resultar con la terapia
**DISENO GENERAL DE LA INVESTIGACION CLINICA:**

Este es un estudio prospectivo, aleatorizado, controlado diseño para comparar dos grupos en la reducción de insuficiencia respiratoria en población con CoViD-19 usando el tratamiento y un grupo de control. Los tratamientos con el gammaCore® se dispensarán de modo profiláctico o agudo. El grupo del control consistirá en pacientes emparejados al grupo de estimulación en la severidad de la indicación y que no serán tratados con el gammaCore®-Sapphire.

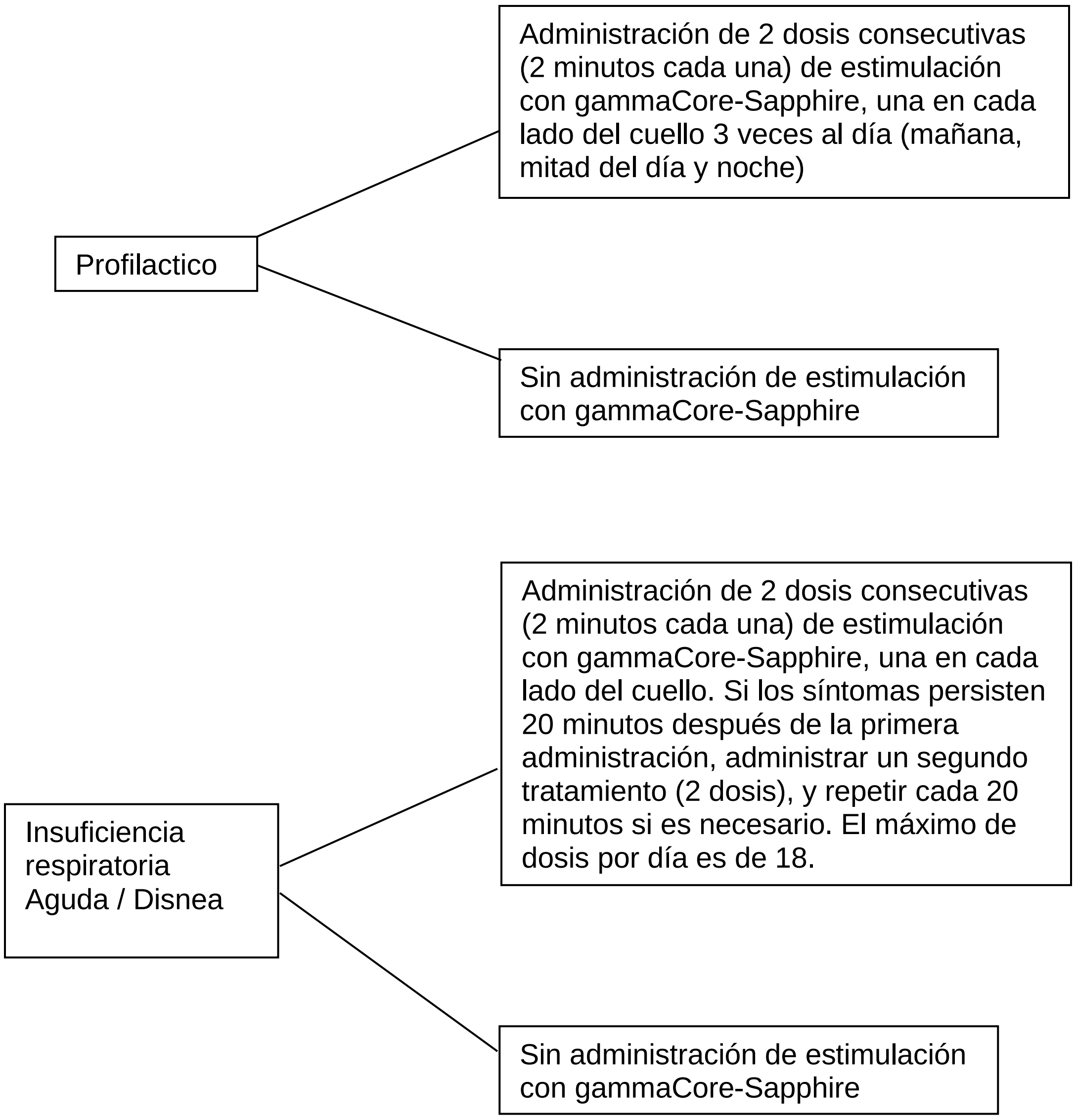

CRITERIOS DE INCLUSION Y EXCLUSION:

Criterios de inclusión - Pacientes que cumplan con todos los criterios enlistados a continuación podrían incluirse en el estudio:

- Paciente mayor de 18 años.
- Paciente ha dado positivo para CoViD-19.
- Paciente con tos y compromiso respiratorio.
- Pacientes con saturación de oxígeno igual o superior a 92% sin necesidad de ventilación mecánica o insuficiencia respiratoria severa que a criterio médico vaya a requerir intubación inmediata.
- El paciente está de acuerdo en usar el dispositivo gammaCore®-Sapphire de acuerdo con las instrucciones y va a seguir los requerimientos del estudio incluyendo el registro de datos requeridos en el estudio.
- El paciente es capaz de proporcionar un consentimiento informado por escrito

Criterios de Exclusión – Pacientes que NO cumpla con alguno los criterios listados a continuación no pueden incluirse en el estudio:

- Mujer embarazada.
- El paciente requiere terapia de oxígeno en casa (i.e. pacientes con EPOC) al inicio del estudio antes del desarrollo de CoVid-19.
- Paciente ya esta inscrito en un ensayo clínico para CoViD-19.
- Paciente con historia de aneurisma, hemorragia intracraneal, tumor cerebral o traumatismo craneal significativo.
- Paciente con enfermedad cardiovascular aterosclerótica grave conocida o sospechada, enfermedad de la arteria carótida grave (p.ej., soplos o antecedentes de ataque isquémico transitorio o accidente cerebrovascular), insuficiencia cardiaca

congestiva, enfermedad arterial coronaria grave conocida o infarto de miocardio reciente.

- Paciente con presión arterial alta, no controlada.
- Pacientes con implantados con un dispositivo eléctrico y / o neuroestimulador, que incluye, entre otros, un marcapasos o desfibrilador cardíaco, neuroestimulador vagal, estimulador cerebral profundo, estimulador espinal, estimulador del crecimiento óseo o implante coclear.
- Pacientes implantados con material metálico de columna cervical o un implante metálico cerca del sitio de estimulación con el gammaCore.
- El paciente pertenece a una población vulnerable o tiene alguna condición que afecta su capacidad de proporcionar consentimiento informado, cumplir con los requisitos de seguimiento o tenga comprometida su capacidad de proporcionar autoevaluaciones (por ejemplo, personas sin hogar, discapacitados física o mental y prisioneros)

PROCEDIMIENTO DE INVESTIGACION CLINICA:

- Inscripción / Día de Admisión
    - Pacientes deben cumplir con todos los criterios de inclusión y ninguno de los criterios de exclusión.
    - Los sujetos deben evaluar y firmar el Consentimiento Informado
    - Recolección de datos demográficos básicos
        - Fecha de nacimiento, género, raza , fumador, región del país.
    - Recolección de medicamentos y comorbilidades
    - Mediciones de Línea Base:
        - Temperatura corporal
        - Presión Arterial
        - Saturación de O2
        - Nivel de Dolor
        - PaO_2/FiO_2

- Disnea/Falta de aliento (Si / No)
- Niveles circulantes de citoquinas:
    - TNF Alfa
    - IL 1 Beta
    - IL 6
- Pro-Calcitonina
- CRP
- Dímero D
- Hemograma con linfocitos absolutos
- Coagulación
- CBC (específicamente células blancas)
- Proteína amiloide sérica a (SAA)
- Ferritina
- Haptoglobina

- Aleatorización
    - Subjeto recibe entrenamiento en el uso del dispositivo gammaCore®-Sapphire

Recolección de Datos

Días 1 - 3
Mediciones de línea base

- Temperatura corporal
- Presión Arterial
- Saturación de O2
- Nivel de Dolor
- PaO_2/FiO_2
- Niveles circulantes de citoquinas:
    - TNF Alfa
    - IL 1 Beta
    - IL 6
- Pro-Calcitonina
- CRP
- Dímero D
- Hemograma con linfocitos absolutos
- Coagulación
- CBC (específicamente células blancas)
- Disnea/Falta de aliento (Si / No)

- Proteína amiloide sérica A
- Ferritina:
- Haptoglobina

Y además

- Número de tratamientos (dosis) de estimulación administrados con el gammaCore®
- Admisión a Unidad de Cuidados Intensivos - UCI (Si / No)
- Necesidad de ventilación (Si / No)
- Supervivencia (Si / No)
- Dado de alta a casa (Si / No)
    - Fecha del alta

A Partir del Día 4 (excepto en los días impares):

- Temperatura corporal
- Presión arterial
- Saturación de O2
- Falta de Aliento (Si / No)
- Número de tratamientos (dosis) de estimulación administrados con gammaCore®
- Necesidad de ventilación (Si / No)
- Supervivencia (Si / No)
- Dado de alta a casa (Si / No)
    - Fecha de alta

Días Impares a Partir del Día 5:

- Igual a Días 1 - 3

NOTA; No se realizarán analíticas adicionales al paciente durante el estudio para la toma de muestras, se tomarán los días que estén marcados por práctica clínica

PUNTOS CLINICOS FINALES DE RENDIMIENTO Y SEGURIDAD

Punto Clínico Final Primario

Reducción de la insuficiencia respiratoria sin la necesidad de utilizar un ventilador mecánico.

Puntos Clínicos Finales Secundarios

Reducción de la mortalidad

Reducción de citoquinas proinflamatorias

Mejoría en el nivel de saturación de O2

Reducción en la necesidad de oxígeno suplementado

Reducción en el tiempo de estadía hospitalaria

Reducción en admisiones a la UCI

Eventos adversos (EA)

ANALISIS PRIMARIOS Y SECUNDARIOS DEL RENDIMIENTO

Análisis Primario de Rendimiento

Se evaluará la tendencia clínica de la necesidad de ventilación mecánica para pacientes con CoViD-19 que reciban estimulación eléctrica no-invasiva del nervio vago con el dispositivo gammaCore®-Sapphire en comparación a un grupo emparejado de control con pacientes que no reciban el tratamiento con estimulación eléctrica.

El porcentaje de pacientes que respondan al tratamiento de estimulación eléctrica no-invasiva del nervio vago con el el gammaCore®-Saphire será comparado con un grupo de control. *Un paciente que responde al tratamiento es aquel* que *no requiere respiración mecánica asistida por un ventilador*.

Análisis Secundario de Rendimiento

Se evaluará la tendencia clínica de la mejoría de los pacientes en términos de mortalidad, admisión en la UCI y el porcentaje de recuperación de pacientes con CoViD-19 que reciban estimulación eléctrica no-invasiva del nervio vago con el dispositivo gammaCore®-Sapphire en comparación a un grupo emparejado de control con pacientes que no reciban el tratamiento con estimulación eléctrica.

Las limitaciones inherentes al tamaño de la muestra serán consideradas en los análisis y análisis adicionales se realizarán apropiadamente.

2 TABLA DE CONTENIDO

1 SYNOPSIS 3
Criterios de inclusión - Pacientes que cumplan con todos los criterios enlistados a continuación podrían incluirse en el estudio: 5
Criterios de Exclusión – Pacientes que NO cumpla con alguno los criterios listados a continuación no pueden incluirse en el estudio: 5
Punto Clínico Final Primario 9
Puntos Clínicos Finales Secundarios 9
2 TABLA DE CONTENIDO 11
3 LISTADO DE ABREVIACIONES Y DEFINICIONES 13
4 INVESTIGADORES CLINICOS Y ESTRUCTURA DE LA ADMINISTRACION DE LA INVESTIGACION CLINICA 15
5 INTRODUCCION 16
5.1 Antecedentes 16
6 OBJETIVOS DE LA INVESTIGACION CLINICA 24
6.1 Objetivo Primario 24
6.2 Objetivo Secundario 24
7 DISEÑO DEL ESTUDIO 25
7.1 Diseño general 25
7.2 Investigación clínica. Definiciones y procedimientos. 26
7.2.1 Calendario de investigación clínica 26
7.2.2 Diagrama de flujo de investigación 29
Tabla 1 Diagrama de flujo de investigación 30
7.3 Justificación del diseño del estudio. 31
7.3.1 Registro de tratamientos y procedimientos previamente administrados y concomitantes. 31
7.4 Selección del tamaño del estudio de investigación clínica 31
7.4.1 Número de participantes 31
7.4.2 Criterios de inclusión 32
7.4.3 Criterios de exclusión 32
7.4.4 Retirada de pacientes del estudio. 33
7.5 Descripción del Sistema médico aplicado en el estudio 34
7.5.1 Identificación y descripción del dispositivo 34
7.5.2 Empaquetado y etiquetado del dispositivo médico. 36
7.5.3 Instalación y uso del Sistema medico 36
7.5.4 Cumplimiento con el uso del dispositivo 37
7.6 Riesgos y beneficios derivados de la participación en el estudio 37
7.7 Puntos finales de rendimiento y seguridad, variables y mediciones 38
7.7.1 Características de los pacientes 38
7.7.2 Puntos Clínicos Finales Primarios y Secundarios 38
Punto Clínico Final Primario 38
Puntos Clínicos Finales Secundarios 38

7.7.3 Variables de rendimiento ... 38
7.7.4 Variables de seguridad ... 39
7.7.5 Otras variables ... 39
7.7.6 Idoneidad de las mediciones con respecto a los objetivos ... 39
7.8 Eventos adversos (EA) y eventos adversos relacionados con el dispositivo (EAD) ... 40
7.8.1 Eventos Adversos/ Definiciones de eventos adversos relacionados con el dispositivo ... 40
7.8.2 Evento adverso grave / Efecto adverso grave del dispositivo / Definiciones de efecto adverso del dispositivo no anticipado ... 41
7.8.3 Informe de eventos adversos / efectos adversos del dispositivo ... 42
7.8.4 Informe de eventos adversos graves / efectos adversos graves del dispositivo ... 44
7.8.5 Deficiencias del dispositivo ... 45
7.9 Aseguramiento de la calidad de los daots ... 45
7.9.1 Monitoreo, auditorias e inspecciones ... 45
7.9.2 Registro de materiales y datos de origen ... 45
7.9.3 Acceso a la fuente de datos y documentación ... 46
7.9.4 Capacitación del personal ... 46
7.9.5 Gestión de datos y calidad ... 46
8 METODOS ESTADISTICOSY DEL TAMAÑO DE LA MUESTRA ... 47
8.1.1 Evaluacion Estadistica de las Variables de Rendimiento y Seguridad ... 47
8.1.2 Manejo de Abandonos, Exclusiones y Datos Perdidos ... 49
8.1.3 Análisis Interino ... 49
8.1.4 Procedures for Reporting any Deviations from the Original Statistical Analysis Plan ... 49
8.2 Enmiendas al Plan de Investigacion Clinica ... 49
8.3 Desviaciones del Plan de Investigación Clínica ... 50
8.4 Reporte y Publicación ... 50
9 ETICA ... 52
9.1 Revisión Comité de Ética ... 52
9.2 Conducta ética de la investigación Clínica ... 52
9.3 Información al sujeto y consentimiento ... 52
9.4 Procedimiento en caso de emergencia médica ... 53
9.5 Protección de datos del sujeto ... 53
9.6 Seguros ... 53
10 BIBLIOGRAFÍA ... 54
11 FIRMAS ... ¡ERROR! MARCADOR NO DEFINIDO.
12 CLINICAL INVESTIGATION PLAN AGREEMENT FORM .. ¡ERROR! MARCADOR NO DEFINIDO.
13 APENDICES ... 56

3 LISTADO DE ABREVIACIONES Y DEFINICIONES

CAP	Ruta colinérgica antinflamatoria
CBC	Conteo total en sangre
CE	Certificación Europea
CEIm	Comité de Ética
CoViD-19	Enfermedad por Coronavirus 2019
CPR	Proteína Reactiva C
CRD	Cuaderno de Recolección de Datos
EA	Evento Adverso
EAD	Efecto Adverso del Dispositivo
EADNA	Evento Adverso del Dispositivo No Anticipado
EANA	Evento Adverso No Anticipado
EASD	Efecto Adverso Grave del Dispositivo
EASDNA	Evento Adverso Grave del Dispositivo No Anticipado
EPOC	Enfermedad Pulmonar Obstructiva Crónica
FDA	Oficina de administración de alimentos y medicamentos de los Estados Unidos
FEV1	Volumen de Expiración Forzado
FiO2	Fracción de oxígeno inhalado
Hz	Hertzios – Unidad de medida de frecuencia
I6	Interleucina 6
IL1-Beta	Interleucina 1-Beta
ITD	Población intención De Tratar
mA	Miliamperios – Unidad de medida de corriente eléctrica
nVNS	Estimulación eléctrica no-invasiva del Nervio Vago
PaO2	Presión parcial arterial de oxigeno
PIC	Plan de Investigación Clínica
PP	Población Por Protocolo
SADR	Síndrome de Deficiencia Respiratoria Aguda

SAE	Evento Adverso Grave
TNF-Alpha	Factor Necrótico Tumoral Alfa
UCI	Unidad de Cuidados Intensivos
VNS	Estimulación eléctrica del Nervio Vago
WOB	Trabajo Respiratorio

4 INVESTIGADORES CLINICOS Y ESTRUCTURA DE LA ADMINISTRACION DE LA INVESTIGACION CLINICA

INVESTIGADOR PRINCIPAL / COORDINADOR DE LA INVESTIGACION

Nombre: Dr. Carlos Tornero MD

Dirección: Hospital Clínico Universitario de Valencia
Segunda Planta – Unidad Reanimación
Avenida Blasco Ibáñez 17
46010 Valencia, España
Teléfono: 610 40 83 81

E-mail: Carlostornero@gmail.com

SITIO DE LA INVESTIGACION CLINICA

Nombre: Hospital Clinico Universitario de Valencia

Dirección: Avenida Blasco Ibáñez 17
46010 Valencia, España
Nombre de Contacto: Dr. Carlos Tornero Tornero

Teléfono: 610 40 83 81

E-mail:
Carlostornero@gmail.com

5 INTRODUCCIÓN

5.1 Antecedentes

La estimulación eléctrica del nervio vago (VNS) tiene una historia previa en la reducción de la insuficiencia respiratoria. Existen al menos dos mecanismos de acción que pueden afectar notablemente la función respiratoria de pacientes con insuficiencia respiratoria debido a la enfermedad CoVID-19.

El primer mecanismo involucra la modulación de la broncoconstricción a través de la VNS.[1,2] La estimulación eléctrica aguda ha demostrado una mejoría acentuada en mediciones del Trabajo Respiratorio (Work of Breathing, WOB) así como en mediciones del Volumen de Expiración Forzado (Forced Expiratory Volume, FEV1) en pacientes con insuficiencia respiratoria severa causada por reactividad de las vías respiratorias. Este efecto parece ocurrir por medio de una respuesta aferente a la estimulación del nervio vago.

El segundo mecanismo, y quizá el más importante, es que se ha demostrado que la VNS es un moderador poderoso de las reacciones inmunes patológicas, específicamente de la supresión de los niveles de citoquinas proinflamatorias a través de la activación de la ruta colinérgica antinflamatoria (Cholinergic Anti-inflammatory Pathway, CAP). En la actualidad, se está estudiando la capacidad de la VNS para modular citoquinas proinflamatorias en una variedad de condiciones inflamatorias agudas y progresivas que comprenden desde el choque séptico y el asma hasta el derrame cerebral, la artritis reumatoidea, y la enfermedad inflamatoria intestinal.[3-7] La VNS se ha estudiado en modelos de choque séptico agudo, demostrando consistentemente su potencial para salvar vidas. En un estudio, una ligación y puntura cecal indujeron un estado séptico en un modelo animal. La VNS redujo la expresión de citoquinas la cual fue asociada estrechamente a la supervivencia.[8] En todos los casos, la terapia con VNS ha demostrado ser una promesa considerable como una alternativa potencial a los esteroides (pues tiene actividad antiinflamatoria sin los efectos negativos asociados con los esteroides) y terapias biológicas que se focalizan en citoquinas inflamatorias (ampliamente, por ejemplo. tofacitinib, o específicamente, adalimumab, etanercept, e infliximab). Mas específicamente, los modelos animales y humanos demuestran que la VNS posee la capacidad para reducir la expresión de mediadores

inflamatorios, incluyendo el factor de necrosis tumoral alfa (TNF-α) y la interleucina-1 beta (IL-1β). Estas son precisamente las citoquinas que presentan niveles elevados en el síndrome de distres respiratorio agudo (SDRA) y otras enfermedades inflamatorias.

Estas razones nos permiten lanzar la hipótesis que la VNS puede mejorar la sobreactividad de la reacción inmune causada por la CoViD-19, confiriendo así una opción terapéutica para aquellos pacientes en la edad de riesgo, o aquellos que estén inmunocomprometidos que podrían llegar a presentar síntomas severos y están en riesgo de desarrollar SDRA.

Estimulación No-Invasiva del Nervio Vago (nVNS)

En el pasado, la VNS se ha proporcionado usando dispositivos eléctricos implantados y acoplados a cables de estimulación con electrodos que se envuelven alrededor del nervio vago. Este nervio es accesible en la vaina carotidea y por tanto es necesaria una cirugía de implantación. Hace poco tiempo la FDA (agencia de alimentos y medicamentos de los Estados Unidos) aprobó el uso de un enfoque terapéutico utilizando la estimulación no-invasiva del nervio vago (nVNS) para el tratamiento y prevención del dolor agudo asociado con dolores de cabeza por migraña y de tipo clúster.

El dispositivo, gammaCore® (electroCore, Inc., Basking Ridge, New Jersey) es portátil y no requiere intervenciones quirúrgicas ni implantables. Un profesional sanitario o el mismo paciente pueden aplicar nVNS externamente usando este dispositivo sobre la piel del cuello, en el área por donde pasa el nervio vago, de manera periódica y dosificada.

En Europa, esta terapia ha recibido el marcado CE para los ***síntomas de enfermedad de las vías respiratorias, incluyendo asma, broncoconstricción, broncoespasmo y la enfermedad pulmonar obstructiva crónica (EPOC),*** el tratamiento y prevención de cefaleas, síntomas de ansiedad y depresión, convulsiones asociadas con epilepsia, desordenes de motilidad gástrica, y los***.***

Específicamente, las indicaciones CE para uso del dispositivo incluyen:

- El tratamiento o prevención de síntomas de enfermedad reactiva de las vías respiratorias, incluyendo asma, broncoconstricción, broncoespasmo inducido por ejercicio, y EPOC.
- El tratamiento agudo y profiláctico de dolor de cabeza primario (migraña, tipo clúster, y hemicránea continua) y dolor de cabeza por sobreuso de fármacos en adultos.

- El uso en adultos como terapia adjunta para la reducción de síntomas de ciertas condiciones de ansiedad y depresión (incluyendo pánico, desorden de estrés postraumático, desorden de obsesión compulsiva, y desorden de depresión mayor).
- Como terapia adjunta en la prevención del comienzo parcial y generalizado de convulsiones asociadas con epilepsia en adultos.
- El uso en adultos como terapia adjunta en el rescate de los síntomas de desórdenes de motilidad gástrica en síndrome de irritabilidad intestinal (incluyendo nausea, vomito, hinchazón/distensión, saciedad temprana y dolor abdominal).

Actualmente se ha propuesto una aprobación de emergencia para usar nVNS con el dispositivo gammaCore en pacientes con CoViD-19 en EEUU.

En relación con la broncoconstricción, estudios clínicos preliminares demostraron que la modulación de la reactividad de las vías respiratorias en pacientes hospitalizados por asma a los que se aplicó nVNS, se obtuvieron mejoras en los datos de permeabilidad de las vías respiratorias.[2,9]
Existen varios estudios con sistemas de VNS implantados y no-invasivos en modelos animales y en humanos que han demostrado la modulación de la cascada inflamatoria y una mejoría en la supervivencia.

La nVNS es un método seguro de estimulación con mínimos efectos secundarios. No requiere intervención quirúrgica ni el uso de procedimientos invasivos que serían limitaciones en casos de pacientes enfermos críticos. La estimulación puede ser administrada por el propio paciente o un profesional sanitario.

El dispositivo gammaCore® para la nVNS se ha estudiado en miles de pacientes en estudios clínicos con un perfil de seguridad excelente y actualmente esta disponible para su uso tanto en los EEUU como en otros países. Además esta introducido como dispositivo de tratamiento a suministrar a pacientes del departamento de defensa y asuntos de veteranos de los Estados Unidos y lleva la marca CE para distribución en Europa.

Los Problemas Respiratorios Asociados con CoViD-19 Involucran la Expresión Severa (Letal) de Citoquinas Desencadenada por la Infección Viral

La enfermedad CoViD-19 (enfermedad por coronavirus 2019) es ocasionada por el SARS-CoV-2 (Severe Acute Respiratory Syndrome Coronavirus 2) y está relacionado con el coronavirus que causo un brote de SARS en el 2003. El virus se transmite por el contacto con microgotas suspendidas en el aire (Ej. tosidos y/o estornudos) o contacto directo con superficies que contienen el virus. El periodo de incubación promedio está en el rango de 2 días a más de dos semanas. Al día 3 de Abril del 2020 se habían confirmado en el mundo más de un millón de casos y alrededor de 50.000 muertes. Esto representa una mortalidad global del 5%, aunque la cantidad base de casos confirmados podría estar marcadamente subestimada. La población de adultos mayores y aquellos con comorbilidades como asma, diabetes, enfermedades cardiacas parecen estar en mas riesgo de morir en casos severos.[10-12]
Muchos pacientes (88%) con CoViD-19 presentan síntomas respiratorios moderados o severos, incluyendo perdida del aliento y disminución en la saturación de oxígeno. Algunos pacientes requieren hospitalización y una minoría significativa progresan severamente hasta ser intubados y necesitar respiración asistida. Debido a la rápida expansión del contagio y la falta de terapias, existen serias preocupaciones en los sistemas de salud internacionales que la cantidad de ventiladores y camas en las UCIs no puedan satisfacer la demanda de casos severos que podrían presentarse en los meses que vienen.

Los pacientes mas afectados pueden desarrollar neumonía y/o síndrome de distrés respiratorio agudo (SDRA o ARDS por sus siglas en inglés). Una de las marcas representativas del SDRA es un incremento drástico en la expresión de citoquinas proinflamatorias, incluyendo TNF-α, IL-1β, IL-6 entre otras. La elevación de niveles de IL-6 parece tener un pronóstico pobre como indicador de compromiso respiratorio. Se cree que la mortalidad a causa del SDRA es al menos parcialmente el resultado de una sobreactividad del sistema inmune del paciente.
Las terapias que pudiesen bloquear la tormenta de citoquinas podrían mejorar la supervivencia y disminuir la necesidad de ventilación mecánica. Otras compañías están, de hecho, desarrollando terapias farmacológicas para mitigar la tormenta de citoquinas (por ejemplo, Cytodyn), o infusiones de plasma proveniente de aquellos pacientes que se han recuperado de la enfermedad. Hoy en día no existe cura o vacuna para protegernos del virus SARS-CoV-2.

<u>Propuesta para Usar nVNS con el dispositivo gammaCore® para Prevenir y/o Reducir la Insuficiencia respiratoria Severa asociada con CoViD-19</u>

Aprovechando la aprobación regulatoria (marca CE) y el excelente perfil de seguridad con el uso de VNS en los últimos treinta años para bloquear la sobreproducción de mediadores inflamatorios a través del CAP (cholinergic anti-inflammatory pathway), y la falta de otras opciones razonables durante esta pandemia de la enfermedad CoViD-19, proponemos poner a disposición de los pacientes, dispositivos gammaCore® para la nVNS en un grupo de pacientes que hayan sido diagnosticados con la enfermedad, pero que no estén en un proceso avanzado del síndrome de insuficiencia respiratoria severa a causa de la tormenta de citoquinas.
La hipotesis es que la administración de la estimulación no-invasiva del nervio vago, usando el dispositivo gammaCore®, durante casos severos de la enfermedad CoViD-19 puede prevenir la respuesta inflamatoria asociada con el proceso agudo de la infección y **disminuir la necesidad de ventilación mecánica y la disminución de la mortalidad por CoViD-19**. **Esperamos que la utilización temprana de la terapia con nVNS en la evolución de la enfermedad pueda reducir la necesidad de utilización de ventilación mecánica y por lo tanto mejorar la supervivencia a través de un enfoque científico seguro y económico.**

ElectroCore® ha diseñado un estimulador no-invasivo del nervio vago llamado gammaCore®. El gammaCore® es un dispositivo portátil, de mano, de fácil uso, y con una batería recargable que produce una señal eléctrica que se aplica sobre un área del cuello próxima al discurrir del nervio vago. Cada dosis de estimulación es relativamente breve (2 minutos) y el usuario mantiene el control de la intensidad de la estimulación.

El dispositivo, gammaCore®, tiene la marca CE y esta comercialmente disponible para el tratamiento de problemas respiratorios como la broncoconstricción, EPOC, el dolor de cabeza por migrañas o tipo clúster, y los desórdenes intestinales, entre otras condiciones. Existe un perfil amplio de seguridad con miles de pacientes tratados en los Estados Unidos y Europa en estudios clínicos y en condiciones de cuidado médico estándar. El Instituto Nacional para la Excelencia Clínica (National Institute for Clinical Excellence, NICE) del Reino Unido le ha otorgado guía favorable para uso en dolor de cabeza tipo clúster.

La aplicación de la terapia con nVNS han sido probados en ensayos clínicos que cuentan con la aprobación de la FDA para el tratamiento del dolor agudo asociado con dolores de cabeza por migraña y episódicos tipo clúster, y la prevención de dolor de cabeza tipo clúster.
Esta experiencia ha demostrado que la nVNS puede administrarse de manera segura con hasta 24 dosis diarias de 2-minutos cada una. **Por lo tanto, existen datos que soportan el método de estimulación del nervio vago de manera eficaz.**
Dado que los modos de acción a través de la modulación del CAP en el tratamiento de la broncoconstricción aguda, el uso de un ejemplo similar para tratar la insuficiencia respiratoria severa asociada con CoViD-19 es razonable. El dispositivo puede utilizarse de forma profiláctica y aguda y en tiempo temprano en el curso de la evolución de la enfermedad antes de la necesidad de ventilación mecánica. Un paciente o un profesional sanitario debidamente entrenado, puede administrar y manejar el tratamiento. Si uno esperase a comenzar la terapia nVNS hasta el momento en que el paciente está bajo ventilación mecánica, es probable que la terapia no pueda ser capaz de bloquear la tormenta de citoquinas y los resultados no serían tan profundos. Se sugiere entonces que la nVNS con el gammaCore® nVNS se haga disponible para el cuidado clínico bajo control en esta experiencia de investigación piloto, y que si la experiencia inicial es positiva, entonces se proceda al diseño de un ensayo clínico más grande.

En términos de los efectos adversos cardíacos y respiratorios teóricos de la estimulación no-invasiva del nervio vago, estos históricamente han incluido bradicardia y broncoconstricción. Se demostró que esto fue el resultado de la estimulación indiscriminada de todas las fibras nerviosas en el nervio vago (este nervio es en su mayoría constituido por fibras tipo A y C). Es posible, sin embargo, disminuir este problema, con la selección de la señal eléctrica de manera que se puedan estimular selectivamente las fibras nerviosas deseadas. Esto es posible porque las fuerzas de campo eléctrico son diferentes para la activación de los diferentes tipos de fibras.[13,14]

La intensidad, duración del pulso y la frecuencia de la función de onda, es decir los parámetros de la señal eléctrica estimulante que proporciona el gammaCore® han sido optimizados para inducir actividad en las fibras grandes mielinizadas tipo Aβ de la rama cervical del nervio vago. Como el gammaCore® solo activa estas fibras Aβ aferentes de bajo umbral en vez de las fibras

tipo C de alto umbral de activación que inervan el corazón, por lo tanto no hay riesgo en relación a sus posibles efectos cardiológicos o por estimulación del sistema parasimpático.

ElectroCore ha conducido estudios preclínicos para evaluar el riesgo potencial de sobreestimulación del nervio vago y sus efectos en el corazón y las vías respiratorias. Varios estudios se hicieron en modelos caninos (Beagles) con las vías respiratorias hipersensibilizadas (peor caso de reactividad de vías respiratorias) con estimulación durante 2 minutos. Los datos de ritmo cardiaco y resistencia en las vías respiratorias antes, durante y después de la estimulación indicaron que no hubo efectos adversos significativos asociados con la estimulación. Estos resultados son consistentes con la experiencia clínica humana usando el dispositivo gammaCore®.

En términos del mecanismo de acción, las fibras aferentes del nervio vago entran al cerebro y hacen sinapsis en el tracto del núcleo solitario (NTS) en el tallo cerebral,[15] y haciendo conexiones con muchas estructuras en el cerebro incluyendo locus coeruleus (LC), la sustancia gris periacueductal (PAG) y el nucleo del rafe magno(RN).[16] Es conocido que estas estructuras controlan el lanzamiento de neurotransmisores inhibitorios claves. En los últimos 25 años, muchos estudios preclínicos y clínicos implican que la actividad de estas estructuras, en particular el LC, es importante en el mecanismo de acción de la estimulación del nervio vago para inhibir convulsiones.

Uso Clínico en este Estudio.

Debido a la falta de otras opciones para los pacientes con alteración respiratoria debido CoViD-19 y la demanda anticipada en nuestros sistemas de salud, el uso de la estimulación no-invasiva del nervio vago con el dispositivo gammaCore® es una alternativa razonable. Los pacientes que entren en el estudio serán observados para determinar si existe un motivo de valor que permita diseñar un ensayo clínico más grande y robusto. Los mejores resultados deberían salir si la nVNS se usa antes de llegar a un estadio más severo de la insuficiencia respiratoria que requiera de ventilación mecánica.

Los mecanismos de broncodilatación y modulación de la tormenta de citoquinas inflamatorias sugieren que una intervención temprana es idónea para mejorar la función pulmonar y evitar la depresión respiratoria. Por tanto, se proponen los siguientes modos de terapia:

1) Profiláctica (preventivo). La modulación de la expresión de citoquinas se puede optimizar con la aplicación de dos dosis consecutivas de dos minutos cada una en cada uno de los lados del cuello tres veces al día. Esto constituye un total de 6 dosis (2 dosis x 3 veces al día) de estimulación en modo profiláctico/preventivo. Esta dosificación es inferior a la dosis máxima diaria (24 dosis por día).
2) Insuficiencia respiratoria aguda: Adicionalmente, y bajo criterio médico, los pacientes que presenten insuficiencia respiratoria aguda o disnea pueden ser estimulados con seis sesiones adicionales de estimulación de dos dosis de dos minutos cada una (es decir, 12 dosis adicionales) en el mismo lado del cuello. Esto ayuda al control sintomático y se puede usar para mejorar el FEV1, el trabajo respiratorio (WOB) o la disnea.

6 OBJETIVOS DE LA INVESTIGACION CLINICA

6.1 Objetivo Primario

- Prevenir la necesidad de un ventilador (respirador) mecánico en pacientes con CoViD-19 mediante la utilización de la estimulación no-invasiva del nervio vago utilizando el dispositivo gammaCore®-Sapphire relativo a un grupo de control.

6.2 Objetivo Secundario

- Evaluar el nivel de citoquinas circulantes. Prevenir las tormentas de citoquinas proinflamatorias.
- Evaluar los requerimientos de oxígeno.
- Disminuir la estancia hospitalaria.
- Disminuir la mortalidad y/o necesidad de cuidados intensivos en pacientes ingresados en el hospital con CoViD-19.
- Medir la frecuencia de eventos adversos que puedan resultar con la terapia.

7 DISEÑO DEL ESTUDIO

7.1 Diseño general

Estudio prospectivo, aleatorizado elaborado sobre 90 pacientes mayores de 18 años con insuficiencia respiratoria secundaria a infección por COVID-19. De forma comparativa se estudiará el efecto que el sistema de tratamiento gammaCore®-Sapphire produce sobre 45 pacientes con insuficiencia respiratoria comparado con 45 pacientes asignados en el grupo control.

Figure 1. Diseño general

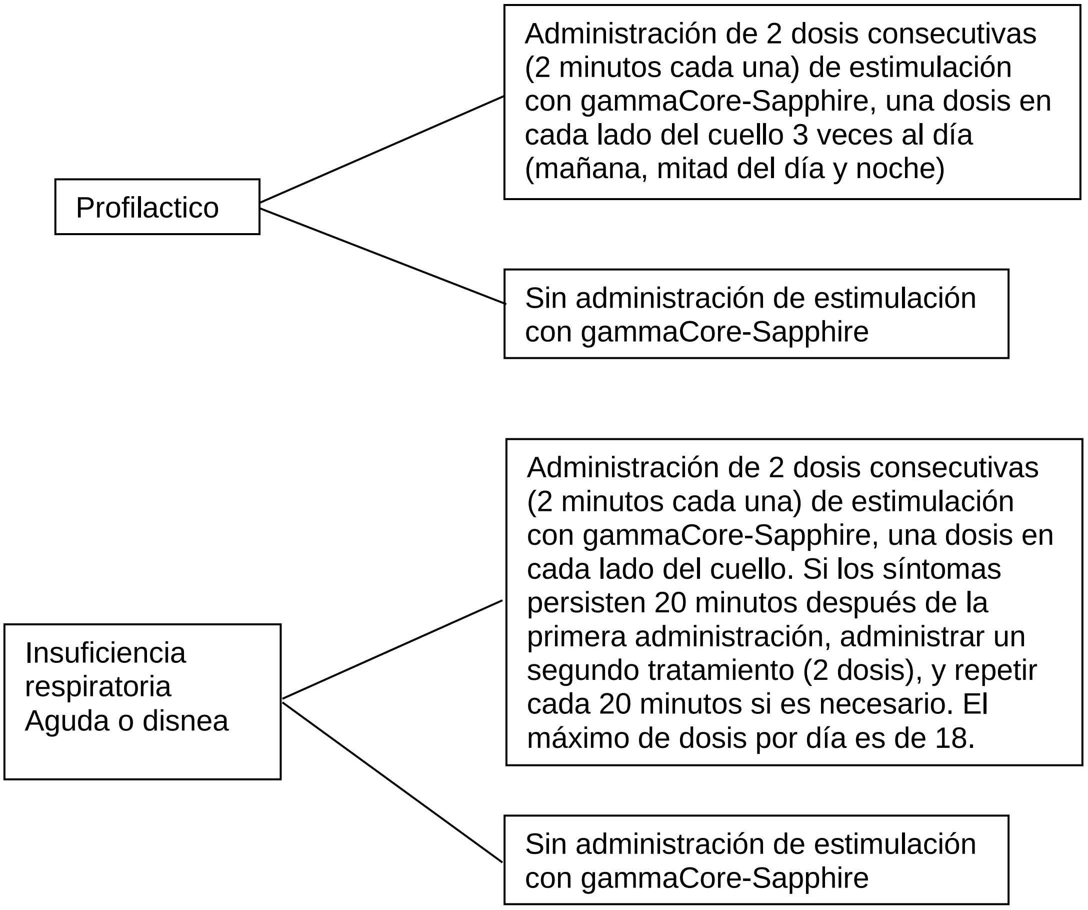

7.2 Investigación clínica. Definiciones y procedimientos.

Tras el diagnóstico del paciente como positivo de COVID y siguiendo las normas y protocolos del hospital se expondrá al paciente el protocolo de tratamiento. El investigador informará al paciente de los procedimientos del estudio y de las condiciones de su participación en el mismo. Tras la firma del consentimiento informado, el paciente será aleatorizado en uno de los dos grupos de tratamiento. Aquellos pacientes asignados en el grupo de gammaCore® serán instruidos en la utilización del dispositivo. El número de usos del dispositivo tanto preventivo como agudo será registrado en el cuaderno de recogida de datos de forma diaria.

7.2.1 Calendario de investigación clínica

Ingreso

Tras el ingreso en el hospital, y una vez el paciente haya sido informado del estudio, firmado el consentimiento, se realizarán las mediciones basales y se clasificarán los pacientes según la gravedad, género y edad.

La gravedad del paciente será clasificada en función de la oxigenación del paciente en:

- Leve
    - cuando la necesidad de FiO2 para mantener una saturación de O2 de 92% sea menor o igual a 0,3
    - PaO2/FiO2 entre 200-300
- Moderada
    - cuando la necesidad de FiO2 para mantener una saturación de O2 de 92% sea de entre 0,3 y 0,5
    - PaO2/FiO2 entre 100-200.
- Grave
    - cuando la necesidad de FiO2 para mantener una saturación de O2 de 92% sea de mayor de 0,5
    - PaO2/FiO2 sea menor o igual a 100

Con el fin de evitar sesgos de asignación, el paciente será asignado al brazo de tratamiento o al brazo control del estudio según la gravedad de la clasificación en una proporción de 1: 1. La aleatorización será balanceada en términos de edad y género.

Para aquellos sujetos asignados al grupo de estimulación no-invasiva del nervio vago por medio del dispositivo gammaCore, el paciente recibirá instrucciones sobre el procedimiento de uso del dispositivo gammaCore®. Se instruirá tanto al paciente como a los profesionales sanitarios encargados del cuidado del paciente en el uso del sistema y en el protocolo, el cual estará disponible a pie de cama.

Protocolo de Administración de la nVNS:

Administrar gammaCore®-Sapphire diariamente, de manera profiláctica, tres veces al día (por la mañana, medio día y noche), aplicando en una sesión 2 dosis consecutivas durante 2 minutos, una en cada lado del cuello. Esto supondría la aplicación de un total de 6 dosis (2 dosis x 3 veces al día).
En caso de insuficiencia respiratoria aguda o disnea hay que aplicar un tratamiento que consiste en 2 dosis consecutivas de 2 minutos cada una. Si la insuficiencia respiratoria o la dificultad para respirar persisten 20 minutos después del inicio del primer tratamiento, se podría administrar una segunda tanda de estimulación (que consiste en 2 dosis consecutivas de otros 2 minutos). El número total de dosis diarias sería 18.

7.2.1.1 Cada día

Las siguientes mediciones deberán ser registrados cada día:

- Temperatura
- Presión arterial
- Saturación O2
- Disnea (Si / No)
- Número de tratamientos (dosis) de estimulación administrados con el gammaCore®
- Admisión a Unidad de Cuidados Intensivos - UCI (Si / No)
- Necesidad de ventilación (Si / No)

- Supervivencia (Si / No)
- Dado de alta a casa (Si / No)
    - Fecha del alta

7.2.1.2 Los días 1,3,5,10,15 y/o al alta

Se deberán registrar los siguientes parámetros:

- PaO_2/FiO_2
- Niveles de citoquinas
    - TNF-Alpha
    - IL-1β
    - IL 6
- Procalcitonina
- CPR
- Dímero D
- Hemograma con linfocitos absolutos
- Coagulación
- CBC (específicamente recuento de células blancas)
- Proteína amiloide sérica A
- Ferritina:
- Haptoglobina.

7.2.1.3 Día de finalización del estudio

El último día del estudio estará determinado por el alta del paciente, la derivación a la unidad de cuidados intensivos (UCI), el inicio de ventilación mecánica o en caso de que el paciente no pueda / no quiera continuar con el estudio. Ese día se registrarán los siguientes datos:

- Temperatura

- Presión arterial
- Saturación O2
- PaO_2/FiO_2
- Niveles de citoquinas
    - TNF-Alpha
    - IL-1β
    - IL 6
- Procalcitonina
- CPR
- Dímero D
- Hemograma con linfocitos absolutos
- Coagulación
- CBC (específicamente recuento de células blancas)
- Proteína amiloide sérica A
- Ferritina:
- Haptoglobina.
- Disnea (Si / No)
- Número de tratamientos (dosis) de estimulación administrados con el gammaCore®
- Admisión a Unidad de Cuidados Intensivos - UCI (Si / No)
- Necesidad de ventilación (Si / No)
- Supervivencia (Si / No)
- Dado de alta a casa (Si / No)
    - Fecha del alta

7.2.2 Diagrama de flujo de investigación

Los procedimientos y medidas de resultado están recopilados en la Tabla 1

Tabla 1 Diagrama de flujo de investigación

Periodo												
Día	D1	D2	D3	D4	D5	D6	D7	D8	D9	D10	D11	D12
Consentimiento informado	✓											
Ingreso	✓											
Criterios de Inclusión /Exclusión	✓					✓						✓
Asignación de dispositivo	✓											
Entrenamiento sobre el dispositivo		✓		✓		✓						
Recogida de datos y registro diario en el CRD						✓						✓
Recopilación de datos médicos						✓						✓
Eventos adversos		✓	✓	✓	✓	✓	✓	✓	✓	✓	✓	✓
Finalización de estudio												✓
Feedback sobre el dispositivo: ¿fue sencillo de usar?						✓						✓

7.3 Justificación del diseño del estudio.

El diseño del estudio en forma aleatorizado con la asignación de un grupo control se sustenta en el objetivo de evaluar la capacidad del dispositivo gammaCore®-Sapphire para mejorar la función respiratoria en pacientes con CoVID-19 positivos en comparación con pacientes con COVID-19 positivos que siguen el tratamiento clínico habitual únicamente.

Durante el periodo de tratamiento en la planta de hospitalización, los pacientes incluidos en el estudio se tratarán mediante el sistema gammaCore® según el protocolo en función de que la administración obedezca a un objetivo profiláctico o a una insuficiencia respiratoria aguda. En caso de que los pacientes no pudiesen aplicarse el tratamiento por si solos, un profesional sanitario les ayudará

El objetivo principal del estudio es evaluar la prevención de ventilación mecánica durante el periodo de hospitalización y la mejoría de la insuficiencia respiratoria con la estimulación eléctrica no-invasiva del nervio vago.

7.3.1 Registro de tratamientos y procedimientos previamente administrados y concomitantes.

El investigador clínico monitorizara el CRD diariamente.
Toda aquella medicación, que se considerase necesaria para la seguridad y el bienestar del paciente, será administrada en función de necesidad siendo comunicado al investigador.
Toda medicación administrada será registrada en el CRD que será rellenado por el investigador de forma diaria.

7.4 Selección del tamaño del estudio de investigación clínica

7.4.1 Número de participantes

Se estudiarán 90 pacientes, 45 en cada grupo de estudio.

7.4.2 Criterios de inclusión

Los sujetos incluidos en el estudio deberán cumplir con todos los criterios de inclusión listados a continuación para poder participar en el estudio de investigación.

1. Pacientes mayores de 18 años
2. Paciente ha dado positivo para CoViD-19
3. Paciente con tos y compromiso respiratorio
4. Pacientes con saturación de oxígeno igual o superior a 92% pero sin necesidad de ventilación mecánica o insuficiencia respiratoria severa que a criterio médico vaya a requerir intubación inmediata.
5. El paciente esta de acuerdo en usar el dispositivo gammaCore®-Sapphire de acuerdo a las instrucciones y va a seguir los requerimientos del estudio incluyendo el registro de datos requeridos en el estudio.
6. El paciente es capaz de proporcionar un consentimiento informado por escrito

7.4.3 Criterios de exclusión

Los sujetos que cumplan cualquiera de los siguientes criterios no pueden incluirse en este estudio de investigación.

1. Mujer embarazada
2. El paciente requiere terapia de oxigeno en casa (i.e. pacientes con EPOC) al inicio del estudio antes del desarrollo de CoVid-19
3. Paciente ya esta inscrito en un ensayo clínico para CoViD-19
4. Paciente con historia de aneurisma, hemorragia intracraneal, tumor cerebral o traumatismo craneal significativo.
5. Paciente con enfermedad cardiovascular aterosclerótica grave conocida o sospechada, enfermedad de la arteria carótida grave (p.ej., soplos o antecedentes

de ataque isquémico transitorio o accidente cerebrovascular), insuficiencia cardiaca congestiva, enfermedad arterial coronaria grave conocida o infarto de miocardio reciente.

6. Paciente con presión arterial alta, no controlada.
7. Pacientes con implantados con un dispositivo eléctrico y / o neuroestimulador, que incluye, entre otros, un marcapasos o desfibrilador cardíaco, neuroestimulador vagal, estimulador cerebral profundo, estimulador espinal, estimulador del crecimiento óseo o implante coclear.
8. Pacientes implantados con material metálico de columna cervical o un implante metálico cerca del sitio de estimulación con el gammaCore.
9. El paciente pertenece a una población vulnerable o tiene alguna condición que afecta su capacidad de proporcionar consentimiento informado, cumplir con los requisitos de seguimiento o tenga comprometida su capacidad de proporcionar autoevaluaciones (por ejemplo, personas sin hogar, discapacitados física o mental y prisioneros)

Cada paciente incluido en el estudio deberá cumplir con todos los siguientes criterios de inclusión y ninguno de los de exclusión

7.4.4 Retirada de pacientes del estudio.

Los pacientes participantes en el estudio serán libres de interrumpir su participación en el estudio en cualquier momento, sin perjuicio de tratamientos adicionales.
Cuando esto suceda el investigador deberá preguntar al paciente los motivos de la retirada voluntaria del estudio y de la existencia de cualquier evento adverso (EA) o efecto adverso relacionado con el dispositivo (EAD) que si fuese el caso, tiene que ser evaluado por un investigador clínico.
Los pacientes podrán ser retirados del estudio en cualquier momento si el Investigador lo considera necesario. Las razones específicas para la retirada de pacientes del estudio son:

- La decisión personal y voluntaria de un paciente de retirada del estudio.

- Evento adverso no aceptable
- Perdida de seguimiento
- El paciente no cumple con los procesos del protocolo del estudio.

En caso de retirada, los EA y EAD deben ser seguidos por los investigadores.
Los pacientes inscritos incorrectamente serán retirados de futuras investigaciones y evaluaciones. Sin embargo, en determinadas circunstancias excepcionales, el sujeto podrá continuar con la investigación, en caso de que la persistencia de la investigación y/ o el seguimiento sean necesarias para la seguridad y el bienestar del paciente, o en caso de que solo quede un período de seguimiento, y que la continuación del sujeto en el estudio no se espera que este asociada con ningún riesgo o molestia adicional para el paciente.

Finalización anticipada del estudio

En caso de que el estudio finalizase de manera anticipada el promotor informará de inmediato a los Investigadores clínicos de la finalización y suspensión y la(s) razón(es) que lo motivan. El Comité de Ética (CEIm) también será informado de inmediato y se le proporcionarán los motivos que condicionaron la finalización de este estudio.

7.5 Descripción del Sistema médico aplicado en el estudio

7.5.1 Identificación y descripción del dispositivo

El gammaCore®-Sapphire, es un estimulador eléctrico percutáneo no invasivo del nervio vago. Es un dispositivo portátil, de sencillo uso que mediante una tecnología moderna y sencilla permite al usuario ajustar la amplitud de la señal. El dispositivo proporciona un sistema de control visual (a través de la pantalla) y audible (mediante pitidos) acerca del dispositivo y el estado de la estimulación.

Consta de un par de superficies de acero inoxidable, que son las que hacen contacto con la piel ("superficies de estimulación"). A través de ellas permite la aplicación de señales eléctricas bajo un mecanismo seguro, patentado y con la aprobación de CE para uso en enfermedades de las vías respiratorias. Previo a la estimulación el paciente aplicará un gel conductor aprobado para este uso sobre las superficies de estimulación en el cuello.

Cabe señalar que las superficies de estimulación del dispositivo deberán están cubiertas cuando el dispositivo no esté en uso.

El gammaCore® desarrolla una señal eléctrica de bajo voltaje que consta de cinco pulsos de 5000 Hz que se repiten a una frecuencia de 25 Hz. La forma de onda de los pulsos eléctricos es sinusoidal con un voltaje máximo limitado a 24 voltios cuando se coloca en la piel y una corriente de salida máxima de 60 mA.

La señal es trasmitida a través de la piel del cuello hasta alcanzar el nervio vago. El gammaCore® permite al paciente posicionar y ajustar adecuadamente el nivel de intensidad de estimulación según las instrucciones de uso.

Cada dosis de estimulación dura 2 minutos, tiempo tras el cual el dispositivo dejará de administrar la estimulación de manera automática. Cada dispositivo permite múltiples tratamientos.

El dispositivo GammaCore® permite administrar hasta treinta dosis de 2 minutos en un período de 24 horas. Una vez haya alcanzado la cantidad máxima diaria de tratamientos, el dispositivo no administrará más estímulos hasta alcanzar el siguiente período de 24 horas.

Con cada dispositivo gammaCore®-Sapphire se incluye una funda de recarga de batería.

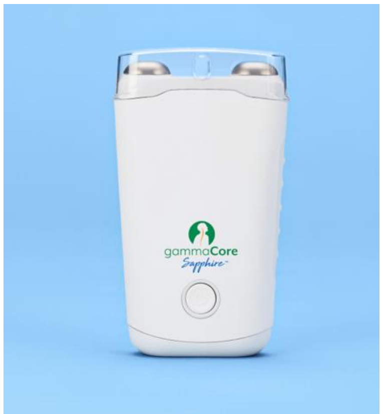

Figura 2. gammaCore®-Sapphire

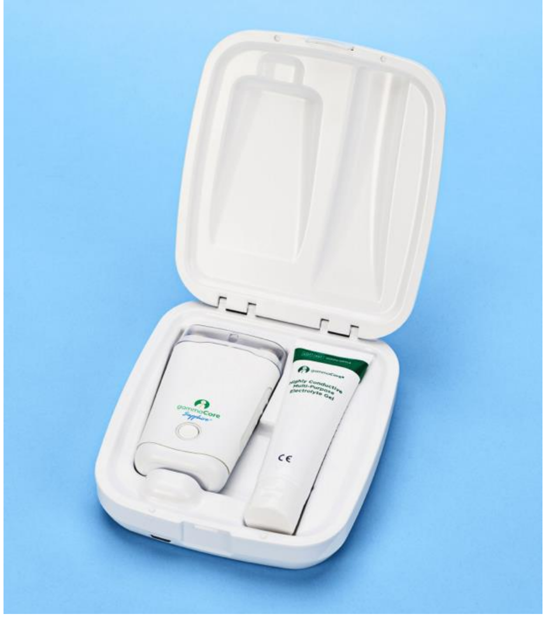

Figura 3. gammaCore®-Sapphire con su funda de recarga

Tras aplicar el gel conductor sobre las superficies de estimulación el paciente o el profesional clínico aplicara el dispositivo sobre la región de la piel del cuello en la que en profundidad se sitúa el nervio vago (entre la tráquea y el músculo esternocleidomastoideo, por encima del pulso carotídeo).

Los detalles específicos sobre la ubicación y el funcionamiento del dispositivo serán proporcionados en las Instrucciones de uso.

7.5.2 Empaquetado y etiquetado del dispositivo médico.

Los dispositivos destinados a esta Investigación clínica serán empaquetados con el embalaje que será marcado como "Solo para Investigación clínica".

7.5.3 Instalación y uso del Sistema medico

El uso del dispositivo será de acuerdo con las instrucciones proporcionadas con el dispositivo. Un miembro específico y cualificado del equipo entrenará a los pacientes participantes.

7.5.4 Cumplimiento con el uso del dispositivo

Todos los usos del dispositivo serán registrados por el equipo de investigación diariamente en el CRD

7.6 Riesgos y beneficios derivados de la participación en el estudio

El dispositivo gammaCore®-Sapphire es un estimulador eléctrico no-invasivo del nervio vago con el marcado CE cuya aplicación y uso se está investigando por medio de este estudio para el tratamiento agudo y profiláctico de la deficiencia respiratoria producida por la infección por CoViD-19. Los beneficios previsibles incluyen:

- Reducir la necesidad de ventilación mecánica.
- Reducción significativa de la estancia hospitalaria.

No existen riesgos significativos identificados y derivados de la participación de los pacientes en este estudio, sin embargo, los pacientes del estudio rara vez pueden experimentar síntomas que en todo caso serían transitorios como:

- Disnea, ronquera o cambio del tono de voz durante el tratamiento.
- Contracciones musculares locales, molestias o dolor transitorio y solo durante la duración de las estimulaciones.
- Sensación de hormigueo, pinchazos o sensación de "alfileres y agujas" en la piel en la que se aplica el dispositivo (parestesia o disestesia).
- Irritación / inflamación de la piel.
- Mareos, desmayo (síncope) durante el tratamiento.
- Cambios en la percepción del gusto (disgeusia). Transitoria

El producto solo se usará en el hospital.

Todos los pacientes deben ser instruidos para su uso antes de comenzar el tratamiento. También se proporcionará instrucciones escritas sobre el uso del dispositivo.

En caso de fallos en el funcionamiento del dispositivo el equipo investigador contactara con el promotor.

Se estima que los beneficios derivados de la participación en este estudio superan a los riesgos, ya que se espera que la mejoría del paciente tratado sea mayor que cualquier efecto secundario posible. El objetivo de este estudio es disminuir la necesidad de ventilación mecánica.

7.7 Puntos finales de rendimiento y seguridad, variables y mediciones

7.7.1 Características de los pacientes

Edad, sexo raza, enfermedades concomitantes

7.7.2 Puntos Clínicos Finales Primarios y Secundarios

Punto Clínico Final Primario

Reducción de la insuficiencia respiratoria sin la necesidad de utilizar un ventilador mecánico.

Puntos Clínicos Finales Secundarios

Reducción de la mortalidad

Reducción de citoquinas proinflamatorias

Mejoría en el nivel de saturación de O2

Reducción en la necesidad de oxígeno suplementado

Reducción en el tiempo de estadía hospitalaria

Reducción en admisiones a la UCI

Eventos adversos (EA)

7.7.3 Variables de rendimiento

Análisis Primario de Rendimiento

Se evaluará la tendencia clínica de la necesidad de ventilación mecánica para pacientes con CoViD-19 que reciban estimulación eléctrica no-invasiva del nervio vago con el dispositivo

gammaCore®-Sapphire en comparación a un grupo emparejado de control con pacientes que no reciban el tratamiento con estimulación eléctrica.

El porcentaje de pacientes que respondan al tratamiento de estimulación eléctrica no-invasiva del nervio vago con el el gammaCore®-Saphire será comparado con un grupo de control. *Un paciente que responde al tratamiento es aquel* que *no requiere respiración mecánica asistida por un ventilador*.

Análisis Secundario de Rendimiento

Se evaluará la tendencia clínica de la mejoría de los pacientes en términos de mortalidad, admisión en la UCI y el porcentaje de recuperación de pacientes con CoViD-19 que reciban estimulación eléctrica no-invasiva del nervio vago con el dispositivo gammaCore®-Sapphire en comparación a un grupo emparejado de control con pacientes que no reciban el tratamiento con estimulación eléctrica.

Las limitaciones inherentes al tamaño de la muestra serán consideradas en los análisis y análisis adicionales se realizarán apropiadamente.

7.7.4 Variables de seguridad

Se evaluará los eventos adversos, su frecuencia y seriedad en base al criterio del investigador. Los eventos adversos relacionados al uso del dispositivo y al dispositivo serán documentados en términos de si son esperados o inesperados.

7.7.5 Otras variables

Se registrarán comorbilidades que se sabe pueden influir en el curso de la enfermedad por CoViD-19, incluyendo asma, bronquitis, enfermedad pulmonar obstructiva crónica u otros trastornos pulmonares subyacentes, diabetes o enfermedades cardíacas.

7.7.6 Idoneidad de las mediciones con respecto a los objetivos

Los objetivos primarios y secundarios incluyen la evaluación de la necesidad de ventilación mecánica y la mortalidad.

También se recogerá y correlacionará estos parámetros con la supresión de citoquinas Es posible que aquellos pacientes que presenten una disminución o no presenten un aumento en la

activación de las citoquinas, presenten una menor necesidad de ventilación mecánica y una mayor supervivencia. Estos parámetros permitirán evaluar los objetivos primarios y secundarios para el estudio.

Las medidas de seguridad son las habituales para un ensayo clínico y permitirán al equipo evaluar la seguridad del dispositivo.

7.8 Eventos adversos (EA) y eventos adversos relacionados con el dispositivo (EAD)

La definición de eventos adversos (EA), efectos adversos del dispositivo (EAD), eventos adversos graves (SAE), efectos adversos graves del dispositivo (EASD) y los mecanismos para su notificación se presentan en las próximas secciones del protocolo. Es de suma importancia que todo el personal investigador esté familiarizado con estas definiciones y los mecanismos de notificación de eventos adversos.

7.8.1 Eventos Adversos/ Definiciones de eventos adversos relacionados con el dispositivo

Eventos Adversos (EA)

Cualquier acontecimiento médico no deseado, enfermedad o lesión no intencional, o signos clínicos adversos (incluidos hallazgos anormales) en sujetos, usuarios u otras personas, estén o no relacionados con el dispositivo médico en investigación.

Nota1: Esta definición incluye eventos relacionados con el dispositivo médico en investigación o el comparador.

Nota 2: Esta definicion incluye eventos relacionados con los procedimientos involucrados

Nota 3: Para los usuarios u otras personas, esta definición está restringida a eventos relacionados con dispositivos médicos en investigación.

Efectos adversos del dispositivo (EAD)

Evento adverso relacionado con el uso de un dispositivo médico en investigación

Nota 1: Esta definición incluye cualquier evento que resulte de insuficiencias o instrucciones inadecuadas para el uso, despliegue, implantación, instalación u operación, o cualquier mal funcionamiento del dispositivo médico en investigación.

Nota 2: Esta definición incluye cualquier evento que sea el resultado de un error del usuario o del uso malintenciondol del dispositivo médico en investigación.

7.8.2 Evento adverso grave / Efecto adverso grave del dispositivo / Definiciones de efecto adverso del dispositivo no anticipado

Evento adverso serio (SAE)

Evento adverso que:

a) Conduce a la muerte
b) Conduce a un grave deterioro en la salud del sujeto que resulta en:
   1. una enfermedad o lesión potencialmente mortal o
   2. un deterioro permanente de una estructura o una función corporales, o
   3. hospitalización prolongada,
   4. intervención médica o quirúrgica para prevenir enfermedades potencialmente mortales o deterioro permanente de una estructura corporal o una función del cuerpo
c) Conduce a sufrimiento fetal, muerte fetal o anormalidad congénita o defecto de nacimiento.

Efecto adverso grave del dispositivo (EASD)

Un efecto adverso grave del dispositivo (EASD) es un efecto del dispositivo no anticipado que resulta en cualquiera de las consecuencias características de un efecto adverso severo o que podría conducir a cualquiera de estas consecuencias si no se toman las medidas adecuadas, si no se realiza una intervención o si las circunstancias son menos oportunas.

Efecto adverso grave no anticipado del dispositivo (EASNAD)

Efectos adversos graves del dispositivo que, por su naturaleza, incidencia, gravedad o resultado, no se han identificado en la versión actual del informe de análisis de riesgos.

Nota: El efecto adverso grave anticipado del dispositivo (EASAD) es un efecto que por naturaleza, incidencia, gravedad o resultado se ha identificado en el informe de análisis de riesgos.

7.8.3 Informe de eventos adversos / efectos adversos del dispositivo

7.8.3.1 Métodos para extraer eventos adversos/efectos adversos del dispositivo

Todos los sujetos serán monitoreados cuidadosamente para detectar eventos adversos durante el período de investigación desde el inicio hasta la finalización del seguimiento. El investigador clínico recopilará información de EA utilizando preguntas no engañosas como "¿ha experimentado algún problema de salud nuevo o empeoramiento de condiciones existentes"? También se registrarán los eventos observados directa o espontáneamente por los sujetos.

Los signos, síntomas y resultados anormales de procedimientos diagnóstico claramente relacionados deben agruparse e informarse como un solo diagnóstico o síndrome siempre que sea posible.

Todos los eventos adversos, incluidos, pero no limitados a los eventos informados por el sujeto o informados en respuesta a una pregunta abierta del investigador clínico o miembro de este equipo, que se incluyen en cualquiera de las definiciones anteriores deben ser reportados como un evento adverso in la forma de investigación clínica y debe incluir la siguiente información.

- Descripción breve del evento (diagnóstico)
- Fecha de inicio (y hora si es relevante)
- Fecha de finalización (y hora si es relevante) (o resolución)
- Gravedad
- Medidas tomadas con respecto al dispositivo médico.

- Opinión sobre causalidad
- Seriedad
- Resultado

Severidad

La gravedad describe la intensidad de un evento y se evaluará como:

Leve

El EA no interfiere de manera significativa con el nivel de funcionamiento normal del sujeto. Puede ser una molestia.

Moderado

El EA produce algún deterioro de la función, pero no es peligroso para la salud. Es incómodo y / o bochornoso.

Grave

El EA produce un deterioro significativo del funcionamiento o incapacidad y / o es un peligro para el sujeto.

Si un EA cambia en severidad, debe ser reportado como un EA de nueva severidad pero con la misma descripción.

Causalidades (Eventos Adversos del Dispositivo, EAD)

La causalidad se evaluará como:

Relacionado (definitivamente, posible o probable)

Una relación causal entre el dispositivo médico clínico en investigación y el EA es al menos una posibilidad razonable, por ejemplo si hay evidencia o argumento que sugiere una relación causal.

No relacionado

No hay indicios de que el EA haya sido causada por el dispositivo médico clínico en investigación.

Los EAD serán reportados en la casilla “si” para EAD en la página del CRD para EA/EAD

Se seguirán los procedimientos descritos para los EA anteriores para documentar los EAD.

7.8.3.2 Seguimiento de sujetos con Eventos Adversos

Cualquier EA que esté en curso cuando el sujeto sea retirado de la investigación debe seguirse hasta que se resuelva la EA o el Investigador Clínico decida que la EA es estable y no necesita más seguimiento. La fecha en que el Investigador clínico considera que se produjo uno de estos resultados para el último EA en curso para un sujeto se considerará la última visita para este sujeto, y el resultado debe registrarse en el CRD.

7.8.4 Informe de eventos adversos graves / efectos adversos graves del dispositivo

Los SAE / EASD se deben informar al Patrocinador dentro de las 24 horas posteriores a la información sobre el evento, independientemente del tiempo que haya transcurrido desde el momento en que ocurrió el evento. El informe inicial debe contener tanta información como sea posible, pero como mínimo la siguiente información:

- Identificación del sujeto
- Especificación del tratamiento
- Diagnóstico del EA
- Especificación del tiempo en que ocurrió el evento medico
- Nombre del Investigador Clínico
- Evaluación de Causalidad
- Criterio de Severidad

El Patrocinador también debe recibir un formulario SAE / SAED completo dentro de los 5 días calendario posteriores a la ocurrencia. Todos los SAE deben informarse, independientemente de si se consideran causalmente relacionados con el dispositivo médico de investigación.

Todos los SAE/ SAED relacionados con el dispositivo médico en investigación estarán sujetos a informes rápidos.

El Promotor debe informar al ECI y autoridades competentes sobre los SAE y SAED asociados con el uso del dispositivo, según los requisitos locales.

7.8.5 Deficiencias del dispositivo

Todas las deficiencias del dispositivo relacionadas con la identidad, calidad, durabilidad, confiabilidad, seguridad o rendimiento de un dispositivo médico en investigación deben documentarse en el CRD por separado.

7.9 Aseguramiento de la calidad de los datos

7.9.1 Monitorización, auditorias e inspecciones

Durante la investigación, el monitor tendrá contactos regulares con el sitio de investigación. Estos contactos incluirán visitas para confirmar que las instalaciones siguen siendo adecuadas para los estándares especificados y que el equipo de investigación está llevando a cabo el procedimiento establecido en el Plan de Investigación Clínica (PIC). Todos los datos deben registrarse con precisión en el CRD. También se realizará la verificación de datos de origen (una comparación de datos en el CRD con los registros médicos del sujeto y otros registros en el sitio de investigación) con acceso a los registros.

El monitor estará disponible entre visitas si el Investigador clínico u otro personal del sitio necesita información y / o asesoramiento.

7.9.2 Registro de materiales y datos de origen

Los datos pueden registrarse directamente en el CRD, que luego se considerará como fuente de datos. Esto debe documentarse en el Manual de monitorización o en una nota de archivo antes de comenzar el estudio.

El origen de los datos fuente en la investigación se especificará más detalladamente en el manual de monitorización ("Origen de los datos fuente").

Es responsabilidad del investigador clínico registrar la información esencial en los registros médicos de acuerdo con los reglamentos y requisitos nacionales, que incluyen:

- Código de Investigación
- Número de examen de sujeto y / o número de sujeto

- Obtención del consentimiento informado para participar en el estudio
- Diagnostico
- Todos los días de entrada de datos durante el período de investigación.
- Todos los EA/EAD
- Tratamientos
- El número de serie del dispositivo.

El investigador clínico es responsable de garantizar la precisión, integridad, legibilidad y puntualidad de los datos registrados en los CRD. Las secciones firmadas de los CRD serán monitorizadas y recolectadas regularmente.

7.9.3 Acceso a la fuente de datos y documentación

El investigador clínico debe garantizar el acceso a los documentos fuente para el monitor y los auditores, así como para la inspección por parte de las agencias reguladoras apropiadas y el IRB / IEC, si es necesario.

7.9.4 Capacitación del personal

El investigador clínico se asegurará de que se brinde la capacitación adecuada relevante para la investigación al personal médico, de enfermería y de otro tipo involucrado y que la nueva información relevante para el desempeño de esta investigación se envíe al personal involucrado.

7.9.5 Gestión de datos y calidad

La herramienta de recopilación de datos para esta prueba será un sistema manual de captura en el CRD. Los datos del sujeto necesarios para el análisis y la presentación de informes se ingresarán en una base de datos o sistema de datos validados. La gestión y el manejo de los datos se llevarán a cabo de acuerdo con el Plan de gestión de datos específico de la investigación de acuerdo con las pautas aplicables y los procedimientos operativos estandar (SOP).
Cualquier desviación, es decir, discrepancias y adiciones al proceso definido en el Plan de gestión de datos se describirá en un informe de Gestión de datos específico de la investigación.

8 METODOS ESTADISTICOSY DEL TAMAÑO DE LA MUESTRA

Considerando un riesgo en el error tipo 1 del 5% ($\alpha = 0.05$) y un riesgo en el error tipo 2 del 20% ($\beta = 0.2$) en una prueba estadística de dos lados y 15% en la tasa de abandono, se estima que se requieren al menos 45 sujetos por grupo para encontrar una diferencia significativa que se esperaría si el 50% de los sujetos en el grupo activo (nVNS con gammaCore) y el 20% de los sujetos en el grupo de control satisfacen el punto clínico final primario del estudio. La aproximación ARCSINUS se usó para calcular el tamaño de la muestra.

8.1.1 Evaluación Estadística de las Variables de Rendimiento y Seguridad

8.1.1.1 Poblaciones de Análisis

Las siguientes poblaciones serán usadas en el análisis estadístico y la presentación de los datos:

- La población para el análisis de seguridad consistirá en todos los sujetos que hayan firmado el consentimiento del estudio y por lo tanto inscritos en el estudio.
- La población de Intención De Tratar (IDT) incluirá los sujetos que cumplan el siguiente criterio:
    - Los sujetos que sean aleatorizados. Para el brazo de tratamiento activo, los sujetos deberán haber usado el dispositivo de acuerdo al protocolo al menos por un día.
- La población Por Protocolo (PP) incluirá sujetos que cumplan el siguiente criterio:
    - Están incluidos en el IDT.
    - No tienen ninguna violación mayor del protocolo que afectan la evaluación de eficacia. Violaciones mayores del protocolo incluyen, pero no están limitadas a lo siguiente:
        - Sujetos en el grupo de cuidado estándar que reciban nVNS
        - Sujetos en grupo de estimulación nVNS que no usen el dispositivo de acuerdo con el protocolo

La línea base y las presentaciones de los resultados de seguridad se basarán en la población de seguridad.

La ITD es considerada la población primaria de análisis, y por tanto se usará para todos los análisis de eficacia primarios y secundarios. Si la diferencia entre la ITD y PP es más del 5%, los análisis de eficacia primarios se repetirán usando la población PP.

Aquellos sujetos que hayan sido candidatos al estudio, pero que no hayan sido incluidos en el estudio no serán considerados en ningún análisis y presentación.

8.1.1.2 Resumen Estadístico

En general, los resultados serán presentados en un resumen estadístico. Los resultados de variables continuas incluirán el número de observaciones, el valor promedio, la desviación estándar, la media, valor máximo, primera cuartilla (Q1), tercera cuartilla (Q3), y valor máximo. Los resultados de variables categóricas se presentarán para cada grupo en términos de porcentajes para cada categoría.

8.1.1.3 Características Demográficas y Otras de Línea Base

La disposición aleatoria de los sujetos, así como las características demográficas (genero, edad, etc.) y otras características de línea base (saturación de O2, nivel de citoquinas, etc.) serán presentadas como parte del resumen estadístico.

El número y porcentaje de sujetos tratados, que hayan completado o abandonado el estudio, y aquellos que hayan sido excluidos de los análisis de rendimiento y seguridad serán tabulados.

8.1.1.4 Grado de Utilización del Dispositivo

La exposición de sujetos en el grupo de nVNS se presentará en el resumen estadístico en término del número de días y el número de dosis diarias.

8.1.1.5 Tratamiento concomitante

Se anotará la información sobre la medicación y terapias concomitantes durante la duración del sujeto en el estudio.

8.1.1.6 Eventos Adversos (EA) y Eventos Adversos del Dispositivo (EAD)

El número total de sujetos con al menos un EA/EAD y el número de EAs/EADs serán computados.

Si hay más de un EA/EAD para un sujeto dentro del mismo periodo de tiempo, estos se contarán como uno solo, reportando aquel con la mayor severidad y relación causal al tratamiento. Los EAs/EADs serán tabulados en términos de severidad y relación casual al tratamiento. En esta tabla, los sujetos con EAs serán identificados con el número del sujeto.

8.1.2 Manejo de Abandonos, Exclusiones y Datos Perdidos

Los valores atípicos no serán utilizados aparte y serán incluidos en los resúmenes y listados. Los datos disponibles de sujetos que hayan salido del estudio de manera prematura serán incluidos en el análisis en la medida de lo posible. Si los datos están parcialmente completos, los que estén disponibles se reportaran dentro de una evaluación de población PP. Las razones por las que cualquier sujeto abandona o es discontinuado de la investigación serán registradas. Si la descontinuación es por motivos de seguridad (o sea eventos adversos), el sujeto será monitorizado hasta que el EA se resuelva o estabilice.

8.1.3 Análisis Interino

El análisis de los puntos clínicos finales del estudio ocurrirá cuando todos los sujetos hayan completado el periodo de hospitalización y los datos hayan sido monitorizados. Como este es el análisis final del objetivo primario del estudio, no habrá ajuste del error de tipo 1.

8.1.4 Procedimientos para reportar cualquier desviación del plan de análisis estadístico original

Cualquier desviación del plan de análisis estadístico original se describirá y ajustará en una enmienda al plan de investigación clínica y/o en un plan de análisis estadístico revisado y/o en el inform final.

8.2 Enmiendas al Plan de Investigacion Clinica

Cualquier cambio al Plan de Investigacion Clinica (PIC) debe ser documentado y justificado por escrito. Cambios al PIC solo seran implementados despues de que un acuerdo escrito se haya alcanzado entre en Investigador Principal/Cooridnador y el Promotor.

La enmienda del PIC requerirá una notificación al Comité de Ética (CEIm). Otros requerimientos locales deberán seguirse si fuese el caso.
El Promotor distribuirá el PIC enmendado al Investigador Principal, quien es responsable de distribuir el documento al CEI y el personal clínico del sitio. La distribución de estos documentos a la Autoridad Competente se manejará de acuerdo a la práctica local.

8.3 Desviaciones del Plan de Investigación Clínica

Se harán todos los esfuerzos para cumplir con los requerimientos del protocolo. Cualquier desviación anticipada del protocolo deberá ser aprobada como un cambio en el PIC antes de que esta proceda. Aprobación del CEIm es necesaria si tal desviación o cambio puede afectar los derechos, seguridad o bienestar de los sujetos. Aquellas desviaciones que se hagan en casos de emergencia para salvaguardar la seguridad y bienestar de los sujetos estarán exentas de aprobación, pero deberán ser notificadas al Promotor y al CEIm tan pronto como sea posible para tomar acciones correctivas. Otro tipo de desviaciones del protocolo, serán documentadas en el registro del estudio con una explicación de la razón. El Promotor es responsable de analizar las desviaciones y evaluar su significancia. Todas las acciones correctivas del caso deberán ser implementadas para evitar desviaciones repetitivas.

8.4 Reporte y Publicación.

Se completará un reporte final de la investigación (Reporte de Investigación Clínica) aun cuando la investigación sea terminada prematuramente. El Promotor preparara el reporte de acuerdo con las guías presentadas en el Anexo C de la normativa ISO 14155-1:2003(E).

Todas las publicaciones y presentaciones se basarán en el Reporte de Investigación Clínica.

Toda la información suministrada por electroCore Inc. en conexión con la investigación permanecerá como propiedad única de electroCore Inc. y será considerada información confidencial. Ninguna información confidencial se podrá compartir con otros sin el consentimiento previo y escrito del Promotor, y no se podrá usar excepto dentro del desarrollo de esta investigación clínica.

El Investigador Principal podrá publicar los resultados de esta investigación, sin embargo, teniendo en cuenta que cierta información relacionada con el dispositivo medico podría ser

estrictamente confidencial, el Promotor tendrá la oportunidad de revisar el manuscrito final antes que este sea sometido para publicación en revistas, conferencias, simposios, u otro tipo de reuniones científicas y no-científicas.

ElectroCore Inc. y su representante líder pueden publicar o presentar los resultados de esta investigación, o cotejar los resultados con los de otros sitios para generar un reporte multicéntrico. Si al Investigador Principal se le ofreciese ser el primer autor, se le delegarán comentar y aprobar la publicación. ElectroCore Inc. y su representante líder tendrán el derecho de usar los resultados para registro, presentaciones internas y promoción.

9 ETICA

9.1 Revisión Comité de Ética

El PIC final, incluida la versión final información del sujeto y consentimiento del sujeto, debe ser aprobado o recibir una opinión favorable por escrito por un Comité de Ética (CEIm) antes de la inscripción de cualquier sujeto en la investigación. El investigador clínico es responsable de informar al CEIm de cualquier enmienda al CIP según los requisitos locales.

9.2 Conducta ética de la investigación Clínica

La investigación se llevará a cabo de conformidad con los requisitos reglamentarios aplicables y con los principios éticos de la última revisión de la Declaración de Helsinki adoptada por la Asociación Médica Mundial (Apéndice A).

9.3 Información al sujeto y consentimiento

Todos los sujetos recibirán información escrita y verbal sobre la investigación antes de cualquier procedimiento relacionado con la misma. Esta información enfatizará que la participación en la investigación es voluntaria y que el sujeto puede retirarse de la investigación en cualquier momento y por cualquier motivo. Todos los sujetos tendrán la oportunidad de hacer preguntas sobre la investigación y se les dará tiempo suficiente para decidir si participar en la investigación.

Antes de cualquier procedimiento relacionado con la investigación, el consentimiento informado será firmado y fechado por el sujeto (o su representante y / o testigo legalmente aceptable, según corresponda) y por el investigador clínico que proporcionó al sujeto la información verbal y escrita.

El consentimiento especifica que los datos se registraran, recopilaran, procesaran y podrán transferirse a otros países en conformidad con la directiva de protección de datos de la Unión Europea (95/46 / CE). Los datos no identificaran a ninguna persona que participe en la investigación.

9.4 Procedimiento en caso de emergencia médica

El investigador clínico es responsable de garantizar que los procedimientos y la experiencia estén disponibles para hacer frente a emergencias médicas durante la investigación.

9.5 Protección de datos del sujeto

La información escrita del sujeto explica que los datos se almacenarán en una base de datos informática, manteniendo la confidencialidad de acuerdo con la legislación nacional de datos, y que los representantes autorizados del Patrocinador, la Autoridad Competente o un CEI requieren acceso directo a esas partes de los registros médicos relevantes para la investigación, incluido el historial médico, para la verificación de datos.

Todos los datos computarizados serán anonimizados y se identificarán solo por número de sujeto.

9.6 Seguros

ElectroCore Inc. tiene un seguro de producto que también cubre la Investigación clínica. Si se requiere de acuerdo con las leyes locales / nacionales, el seguro se suscribirá para ese país específico.

10 BIBLIOGRAFÍA

Se adjunta la siguiente bibliografía con el objetivo de poner en contexto la situación clínica y exponer los riesgos y beneficios derivados del uso del dispositivo.
Las referencias abajo citadas han sido revisadas y evaluadas para constituir la sección de antecedentes del proyecto.

1. Hoffmann TJ, Mendez S, Staats P, Emala CW, Guo P. Inhibition of histamine-induced bronchoconstriction in Guinea pig and Swine by pulsed electrical vagus nerve stimulation. Neuromodulation. 2009;12(4):261–269.
2. Steyn E, Mohamed Z, Husselman C. Non-invasive vagus nerve stimulation for the treatment of acute asthma exacerbations-results from an initial case series. Int J Emerg Med. 2013;6(1):7.
3. Pavlov VA, Chavan SS, Tracey KJ. Bioelectronic medicine: from preclinical studies on the inflammatory reflex to new approaches in disease diagnosis and treatment. Cold Spring Harb Perspect Med. 2020;10(3):a034140.
4. Koopman FA, Chavan SS, Miljko S, et al. Vagus nerve stimulation inhibits cytokine production and attenuates disease severity in rheumatoid arthritis. Proc Natl Acad Sci U S A. 2016;113(29):8284–8289.
5. Brock C, Brock B, Aziz Q, et al. Transcutaneous cervical vagal nerve stimulation modulates cardiac vagal tone and tumor necrosis factor-alpha. Neurogastroenterol Motil. 2017;29(5):10.1111/nmo.12999.
6. Tarn J, Legg S, Mitchell S, Simon B, Ng WF. The effects of noninvasive vagus nerve stimulation on fatigue and immune responses in patients with primary sjögren's syndrome. Neuromodulation. 2019;22(5):580–585.
7. Lerman I, Hauger R, Sorkin L, et al. Noninvasive transcutaneous vagus nerve stimulation decreases whole blood culture-derived cytokines and chemokines: A randomized, blinded, healthy control pilot trial. Neuromodulation. 2016;19(3):283–290.
8. Huston JM, Gallowitsch-Puerta M, Ochani M, et al. Transcutaneous vagus nerve stimulation reduces serum high mobility group box 1 levels and improves survival in murine sepsis. Crit Care Med. 2007;35(12):2762–2768.
9. Miner JR, Lewis LM, Mosnaim GS, Varon J, Theodoro D, Hoffmann TJ. Feasibility of percutaneous vagus nerve stimulation for the treatment of acute asthma exacerbations. Acad Emerg Med. 2012;19(4):421–429.
10. Cao Y, Liu X, Xiong L, Cai K. Imaging and Clinical Features of Patients With 2019 Novel Coronavirus SARS-CoV-2: A systematic review and meta-analysis [published online ahead of print, 2020 Apr 3]. J Med Virol. 2020;10.1002/jmv.25822.
11. Zhou M, Zhang X, Qu J. Coronavirus disease 2019 (COVID-19): a clinical update [published online ahead of print, 2020 Apr 2]. Front Med. 2020;10.1007/s11684-020-0767-8.
12. Chatterjee P, Nagi N, Agarwal A, et al. The 2019 novel coronavirus disease (COVID-19) pandemic: A review of the current evidence [published online ahead of print, 2020 Mar 30]. Indian J Med Res. 2020;10.4103/ijmr.IJMR_519_20.
13. Zhu YF, Wu Q, Henry JL. Changes in functional properties of A-type but not C-type sensory neurons in vivo in a rat model of peripheral neuropathy. J Pain Res. 2012;5:175–192. doi:10.2147/JPR.S26367.
14. McAllen RM, Shafton AD, Bratton BO, Trevaks D, Furness JB. Calibration of thresholds for functional engagement of vagal A, B and C fiber groups *in vivo*. *Bioelectron Med (Lond)*. 2018;1(1):21–27.

15. Zoccal DB, Furuya WI, Bassi M, Colombari DS, Colombari E. The nucleus of the solitary tract and the coordination of respiratory and sympathetic activities. Front Physiol. 2014;5:238.
16. Ruffoli R, Giorgi FS, Pizzanelli C, Murri L, Paparelli A, Fornai F. The chemical neuroanatomy of vagus nerve stimulation. J Chem Neuroanat. 2011;42(4):288–296.

11 APENDICES

11.1 Declaración de Helsinki

DECLARACIÓN DE LA ASOCIACIÓN MÉDICA MUNDIAL DE HELSINKI
Principios éticos para la investigación médica en humanos
Adoptada por la 18a Asamblea General de la AMM, Helsinki, Finlandia, junio de 1964, y enmendada por:
29a Asamblea General de la AMM, Tokio, Japón, octubre de 1975
35ª Asamblea General de la AMM, Venecia, Italia, octubre de 1983
41a Asamblea General de la AMM, Hong Kong, septiembre de 1989
48ª Asamblea General de la AMM, Somerset West, República de Sudáfrica, octubre de 1996
52ª Asamblea General de la AMM, Edimburgo, Escocia, octubre de 2000
53ª Asamblea General de la AMM, Washington 2002 (Nota de aclaración sobre el párrafo 29 agregado)
55ª Asamblea General de la AMM, Tokio 2004 (se agregó una nota de aclaración sobre el párrafo 30)
59ª Asamblea General de la AMM, Seúl, octubre de 2008
64ª Asamblea General de la AMM, Fortaleza, Brasil, octubre de 2013

Preámbulo

1. La Asociación Médica Mundial (AMM) ha desarrollado la Declaración de Helsinki como una declaración de principios éticos para la investigación médica en seres humanos, incluida la investigación sobre material y datos humanos identificables.
La Declaración está destinada a leerse en su conjunto y cada uno de sus párrafos constituyentes no debe aplicarse sin tener en cuenta todos los demás párrafos relevantes.
2. De conformidad con el mandato de la AMM, la Declaración está dirigida principalmente a los médicos. La AMM alienta a otros que están involucrados en investigaciones médicas que involucran sujetos humanos a adoptar estos principios.

Principios generales

3. La Declaración de Ginebra de la AMM vincula al médico con las palabras: "La salud de mi paciente será mi primera consideración", y el Código Internacional de Ética Médica declara que "un médico actuará en el mejor interés del paciente cuando proporcionando atención médica ".
4. Es deber del médico promover y salvaguardar la salud, el bienestar y los derechos de los pacientes, incluidos los que participan en la investigación médica. El conocimiento y la conciencia del médico están dedicados al cumplimiento de este deber.
5. El progreso médico se basa en investigaciones que finalmente deben incluir estudios que involucren sujetos humanos.
6. El objetivo principal de la investigación médica en seres humanos es comprender las causas, el desarrollo y los efectos de las enfermedades y mejorar las intervenciones preventivas, diagnósticas y terapéuticas (métodos, procedimientos y tratamientos). Incluso las mejores intervenciones actuales deben evaluarse continuamente a través de la investigación por su seguridad, efectividad, eficiencia, accesibilidad y calidad.

7. La investigación médica está sujeta a estándares éticos que promueven y garantizan el respeto de todos los seres humanos y protegen su salud y sus derechos.
8. Si bien el objetivo principal de la investigación médica es generar nuevo conocimiento, este objetivo nunca puede tener prioridad sobre los derechos e intereses de los sujetos de investigación individuales.
9. Es deber de los médicos que participan en la investigación médica proteger la vida, la salud, la dignidad, la integridad, el derecho a la libre determinación, la privacidad y la confidencialidad de la información personal de los sujetos de investigación. La responsabilidad de la protección de los sujetos de investigación siempre debe recaer en el médico u otros profesionales de la salud y nunca con los sujetos de investigación, a pesar de que hayan dado su consentimiento.
10. Los médicos deben tener en cuenta las normas y estándares éticos, legales y reglamentarios para la investigación con seres humanos en sus propios países, así como las normas y estándares internacionales aplicables. Ningún requisito ético, legal o reglamentario nacional o internacional debe reducir o eliminar ninguna de las protecciones para los sujetos de investigación establecidos en esta Declaración.
11. La investigación médica debe llevarse a cabo de manera que minimice el posible daño al medio ambiente.
12. La investigación médica en seres humanos debe ser realizada solo por personas con la ética adecuada y educación científica, capacitación y calificaciones. La investigación en pacientes o voluntarios sanos requiere la supervisión de un médico competente y debidamente calificado u otro profesional de la salud.
13. Los grupos que están subrepresentados en la investigación médica deben tener acceso apropiado para participar en la investigación.
14. Los médicos que combinan la investigación médica con la atención médica deben involucrar a sus pacientes en la investigación solo en la medida en que esto se justifique por su potencial valor preventivo, diagnóstico o terapéutico y si el médico tiene buenas razones para creer que la participación en el estudio de investigación no afectar negativamente la salud de los pacientes que sirven como sujetos de investigación.
15. Debe garantizarse la compensación y el tratamiento adecuados para los sujetos perjudicados como resultado de participar en la investigación.
Riesgo, cargas y beneficios
16. En la práctica médica y en la investigación médica, la mayoría de las intervenciones implican riesgos y cargas.
La investigación médica en seres humanos solo puede realizarse si la importancia del objetivo supera los riesgos y las cargas para los sujetos de investigación.
17. Toda investigación médica que involucre sujetos humanos debe ser previa
medido por una evaluación cuidadosa de riesgos y cargas predecibles para los individuos y grupos involucrados en la investigación en comparación con los beneficios previsibles para ellos y para otros individuos o grupos afectados por la condición bajo investigación.
Se deben implementar medidas para minimizar los riesgos. Los riesgos deben ser continuamente monitoreados, evaluados y documentados por el investigador.

18. Es posible que los médicos no participen en un estudio de investigación con seres humanos a menos que estén seguros de que los riesgos se han evaluado adecuadamente y se pueden manejar de manera satisfactoria.
Cuando se descubre que los riesgos superan los beneficios potenciales o cuando hay pruebas concluyentes de resultados definitivos, los médicos deben evaluar si continuar, modificar o suspender inmediatamente el estudio.

Grupos e individuos vulnerables

19. Algunos grupos e individuos son particularmente vulnerables y pueden tener una mayor probabilidad de ser perjudicados o de sufrir daños adicionales.
Todos los grupos e individuos vulnerables deben recibir protección específicamente considerada.
20. La investigación médica con un grupo vulnerable solo se justifica si la investigación responde a las necesidades de salud de las prioridades de este grupo y la investigación no puede llevarse a cabo en un grupo no vulnerable. Además, este grupo debería beneficiarse del conocimiento, las prácticas o las intervenciones que resultan de la investigación.

Requisitos científicos y protocolos de investigación

21. La investigación médica en seres humanos debe ajustarse a los principios científicos generalmente aceptados, basarse en un conocimiento exhaustivo de la literatura científica, otras fuentes de información relevantes y una experimentación adecuada en laboratorio y, según corresponda, con animales. Se debe respetar el bienestar de los animales utilizados para la investigación.
22. El diseño y el desempeño de cada estudio de investigación que involucre sujetos humanos debe describirse claramente y justificarse en un protocolo de investigación.
El protocolo debe contener una declaración de las consideraciones éticas involucradas y debe indicar cómo se han abordado los principios de esta Declaración. El protocolo debe incluir información sobre financiación, promotores, afiliaciones institucionales, posibles conflictos de intereses, incentivos para los sujetos e información sobre disposiciones para tratar y / o compensar a los sujetos perjudicados como consecuencia de la participación en el estudio de investigación.
En ensayos clínicos, el protocolo también debe describir las disposiciones apropiadas para las disposiciones posteriores al juicio.

Comités de ética de investigación

23. El protocolo de investigación debe enviarse para su consideración, comentario, orientación y aprobación al comité de ética de investigación correspondiente antes de que comience el estudio. Este comité debe ser transparente en su funcionamiento, debe ser independiente del investigador, el promotor y cualquier otra influencia indebida y debe estar debidamente calificado. Debe tener en cuenta las leyes y reglamentos del país de los países en los que se realizará la investigación, así como las normas y estándares internacionales aplicables, pero no debe permitirse que reduzcan o eliminen ninguna de las protecciones para los sujetos de investigación establecidas en esta Declaración
El comité debe tener el derecho de monitorear los estudios en curso. El investigador debe proporcionar información de seguimiento al comité, especialmente información sobre cualquier evento adverso grave. No se pueden realizar enmiendas al protocolo sin la consideración y

aprobación del comité. Después del final del estudio, los investigadores deben presentar un informe final al comité que contenga un resumen de los hallazgos y conclusiones del estudio.

Privacidad y confidencialidad

24. Se deben tomar todas las precauciones para proteger la privacidad de los sujetos de investigación y la confidencialidad de su información personal.

Consentimiento informado

25. La participación de individuos capaces de dar su consentimiento informado como sujetos en investigación médica debe ser voluntaria. Aunque puede ser apropiado consultar a miembros de la familia o líderes de la comunidad, ninguna persona capaz de dar su consentimiento informado puede inscribirse en un estudio de investigación a menos que él o ella lo acepte libremente.

26. En la investigación médica que involucra sujetos humanos capaces de dar su consentimiento informado, cada sujeto potencial debe estar adecuadamente informado de los objetivos, métodos, fuentes de financiamiento, posibles conflictos de interés, afiliaciones institucionales del investigador, los beneficios anticipados y los riesgos potenciales de el estudio y la incomodidad que pueda conllevar, disposiciones posteriores al estudio y cualquier otro aspecto relevante del estudio. El sujeto potencial debe ser informado del derecho de negarse a participar en el estudio o de retirar su consentimiento para participar en cualquier momento sin represalias. Se debe prestar especial atención a las necesidades específicas de información de sujetos potenciales individuales, así como a los métodos utilizados para entregar la información.

Después de asegurarse de que el sujeto potencial ha entendido la información, el médico u otra persona debidamente calificada debe buscar el consentimiento informado otorgado libremente por el sujeto potencial,

preferiblemente por escrito. Si el consentimiento no puede expresarse por escrito, el consentimiento no escrito debe documentarse y atestiguarse formalmente.

Todos los sujetos de investigación médica deben tener la opción de ser informados sobre el resultado general y los resultados del estudio.

27. Al buscar el consentimiento informado para participar en un estudio de investigación, el médico debe ser particularmente cauteloso si el sujeto potencial está en una relación dependiente con el médico o puede consentir bajo presión. En tales situaciones, el consentimiento informado debe ser solicitado por un individuo debidamente calificado que sea completamente independiente de esta relación.

28. Para un sujeto de investigación potencial que es incapaz de dar su consentimiento informado, el médico debe buscar el consentimiento informado del representante legalmente autorizado. Estas personas no deben incluirse en un estudio de investigación que no tenga ninguna probabilidad de beneficio para ellos, a menos que esté destinado a promover la salud del grupo representado por el sujeto potencial, la investigación no puede realizarse con personas capaces de dar su consentimiento informado, y La investigación implica solo un riesgo mínimo y una carga mínima.

29. Cuando un sujeto de investigación potencial que se considera incapaz de dar su consentimiento informado puede dar su consentimiento a las decisiones sobre su participación en la investigación, el médico debe buscar ese consentimiento además del consentimiento del representante legalmente autorizado. La disidencia del sujeto potencial debe ser respetada.

30. La investigación que involucra a sujetos que son física o mentalmente incapaces de dar su consentimiento, por ejemplo, pacientes inconscientes, se puede hacer solo si la condición física o mental que impide dar el consentimiento informado es una característica necesaria del grupo de investigación. En tales circunstancias, el médico debe buscar el consentimiento informado del representante legalmente autorizado. Si dicho representante no está disponible y si la investigación no puede retrasarse, el estudio puede continuar sin el consentimiento informado, siempre que las razones específicas para involucrar a los sujetos con una condición que los haga incapaces de dar el consentimiento informado se hayan indicado en el protocolo de investigación y el estudio. ha sido aprobado por un comité de ética de investigación. El consentimiento para permanecer en la investigación debe obtenerse lo antes posible del sujeto o de un representante legalmente autorizado.

31. El médico debe informar completamente al paciente qué aspectos de su atención están relacionados con la investigación. La negativa de un paciente a participar en un estudio o la decisión del paciente de retirarse del estudio nunca debe afectar negativamente la relación médico-paciente.

32. Para la investigación médica que utiliza material o datos humanos identificables, como la investigación de material o datos contenidos en biobancos o depósitos similares, los médicos deben buscar el consentimiento informado para su recopilación, almacenamiento y / o reutilización. Puede haber situaciones excepcionales donde el consentimiento sería imposible o impracticable para obtener dicha investigación. En tales situaciones, la investigación puede realizarse solo después de la consideración y aprobación de un comité de ética de investigación.

Uso de placebo

33. Los beneficios, riesgos, cargas y efectividad de una nueva intervención deben ser probados contra los de la (s) mejor (es) intervención (es) probada (s), excepto en las siguientes circunstancias:

Donde no existe una intervención comprobada, el uso de placebo, o ninguna intervención, es aceptable

Donde, por razones metodológicas convincentes y científicamente sólidas, el uso de cualquier intervención menos efectiva que la mejor probada, el uso de placebo o ninguna intervención es necesaria para determinar la eficacia o seguridad de una intervención

y los pacientes que reciben cualquier intervención menos efectiva que la mejor probada, placebo o ninguna intervención no estarán sujetos a riesgos adicionales de daños graves o irreversibles como resultado de no recibir la mejor intervención probada.

Se debe tener mucho cuidado para evitar el abuso de esta opción.

Disposiciones posteriores al juicio

34. Antes de un ensayo clínico, los promotores, los investigadores y los gobiernos del país anfitrión deberían tomar medidas para el acceso posterior al ensayo para todos los participantes que aún necesitan una intervención identificada como beneficiosa en el ensayo. Esta información también debe divulgarse a los participantes durante el proceso de consentimiento informado.

Registro de investigación y publicación y difusión de resultados

35. Todos los estudios de investigación que involucren sujetos humanos deben registrarse en una base de datos de acceso público antes del reclutamiento del primer sujeto.

36. Investigador, autores, promotores, editores y editores tienen obligaciones éticas con respecto a la publicación y difusión de los resultados de la investigación. Los investigadores tienen el deber de poner a disposición del público los resultados de su investigación sobre sujetos humanos y son responsables de la integridad y precisión de sus informes. Todas las partes deben cumplir con las pautas aceptadas para la presentación de informes éticos. Los resultados negativos y no concluyentes, así como los positivos, deben publicarse o ponerse a disposición del público. Fuentes de financiamiento, institución Todas las afiliaciones y conflictos de intereses deben declararse en la publicación. Los informes de investigación que no estén de acuerdo con los principios de esta Declaración no deben aceptarse para su publicación.

Intervenciones no comprobadas en la práctica clínica

37. En el tratamiento de un paciente individual, donde no existen intervenciones comprobadas u otras intervenciones conocidas han sido ineficaces, el médico, después de buscar el asesoramiento de expertos, con el consentimiento informado del paciente o un representante legalmente autorizado, puede utilizar una intervención no probada si a juicio del médico, ofrece la esperanza de salvar vidas, restablecer la salud o aliviar el sufrimiento. Posteriormente, esta intervención debe ser objeto de investigación, diseñada para evaluar su seguridad y eficacia. En todos los casos, la nueva información debe registrarse y, cuando corresponda, ponerse a disposición del público.
